# Design and Characterization of a Human Monoclonal Antibody that Modulates Mutant Connexin 26 Hemichannels Implicated in Deafness and Skin Disorders

**DOI:** 10.3389/fnmol.2017.00298

**Published:** 2017-09-22

**Authors:** Liang Xu, Andrea Carrer, Francesco Zonta, Zhihu Qu, Peixiang Ma, Sheng Li, Federico Ceriani, Damiano Buratto, Giulia Crispino, Veronica Zorzi, Gaia Ziraldo, Francesca Bruno, Chiara Nardin, Chiara Peres, Flavia Mazzarda, Anna M. Salvatore, Marcello Raspa, Ferdinando Scavizzi, Youjun Chu, Sichun Xie, Xuemei Yang, Jun Liao, Xiao Liu, Wei Wang, Shanshan Wang, Guang Yang, Richard A. Lerner, Fabio Mammano

**Affiliations:** ^1^Shanghai Institute for Advanced Immunochemical Studies, ShanghaiTech University Shanghai, China; ^2^Institute of Biochemistry and Cell Biology, Shanghai Institute for Biological Sciences, Chinese Academy of Sciences Shanghai, China; ^3^CNR Institute of Cell Biology and Neurobiology Monterotondo, Italy; ^4^Department of Physics and Astronomy “G. Galilei,”, University of Padova Padova, Italy; ^5^Venetian Institute of Molecular Medicine Padova, Italy; ^6^Institute of Otolaryngology, Catholic University School of Medicine Rome, Italy; ^7^Department of Science, Roma Tre University Rome, Italy; ^8^School of Life Science and Technology, Shanghai Tech University Shanghai, China; ^9^University of Chinese Academy of Sciences Beijing, China; ^10^Department of Cell and Molecular Biology, The Scripps Research Institute La Jolla, CA, United States

**Keywords:** syndromic autosomal hereditary deafness, cochlea, keratinocytes, connexins, antibody library, antibody structure, epitope identification

## Abstract

**Background:** Mutations leading to changes in properties, regulation, or expression of connexin-made channels have been implicated in 28 distinct human hereditary diseases. Eight of these result from variants of connexin 26 (Cx26), a protein critically involved in cell-cell signaling in the inner ear and skin. Lack of non-toxic drugs with defined mechanisms of action poses a serious obstacle to therapeutic interventions for diseases caused by mutant connexins. In particular, molecules that specifically modulate connexin hemichannel function without affecting gap junction channels are considered of primary importance for the study of connexin hemichannel role in physiological as well as pathological conditions. Monoclonal antibodies developed in the last three decades have become the most important class of therapeutic biologicals. Recombinant methods permit rapid selection and improvement of monoclonal antibodies from libraries with large diversity.

**Methods:** By screening a combinatorial library of human single-chain fragment variable (scFv) antibodies expressed in phage, we identified a candidate that binds an extracellular epitope of Cx26. We characterized antibody action using a variety of biochemical and biophysical assays in HeLa cells, organotypic cultures of mouse cochlea and human keratinocyte-derived cells.

**Results:** We determined that the antibody is a remarkably efficient, non-toxic, and completely reversible inhibitor of hemichannels formed by connexin 26 and does not affect direct cell-cell communication via gap junction channels. Importantly, we also demonstrate that the antibody efficiently inhibits hyperative mutant Cx26 hemichannels implicated in autosomal dominant non-syndromic hearing impairment accompanied by keratitis and hystrix-like ichthyosis-deafness (KID/HID) syndrome. We solved the crystal structure of the antibody, identified residues that are critical for binding and used molecular dynamics to uncover its mechanism of action.

**Conclusions:** Although further studies will be necessary to validate the effect of the antibody *in vivo*, the methodology described here can be extended to select antibodies against hemichannels composed by other connexin isoforms and, consequently, to target other pathologies associated with hyperactive hemichannels. Our study highlights the potential of this approach and identifies connexins as therapeutic targets addressable by screening phage display libraries expressing human randomized antibodies.

## Introduction

Connexin-made channels are essential and widespread constituents of the cell-cell communication pathways that enable the direct exchange of nutrients and signaling molecules between adjacent cells (Goodenough and Paul, 2009) or between cell cytoplasm and the extracellular milieu (Saez and Leybaert, 2014). Channel constituents are structurally homogeneous plasma membrane proteins encoded by twenty one connexin genes in the human genome (http://www.genenames.org/cgi-bin/genefamilies/set/314). Connexin proteins share the same topology that comprises four transmembrane domains (TM1–TM4) connected by two extracellular loops (EC1 and EC2) and a cytoplasmic loop (CL), while the N- and C-termini (NT, CT) of the protein extend into the cytoplasm of the cell (Maeda et al., 2009). Connexins are post-translationally oligomerized to form hexameric assemblies, known as connexons or hemichannels, prior to membrane insertion either within the endoplasmic reticulum or in the trans-Golgi network (Esseltine and Laird, 2016). Connexin hemichannels are then trafficked to the plasma membrane, where the head-to-head docking of two hemichannels from adjacent cells promotes the formation of an intercellular (gap junction) channel. These channels are characterized by an aqueous pore with a diameter typically >1 nm (Maeda et al., 2009; Esseltine and Laird, 2016; Zong et al., 2016), and are permeable to several current-carrying anions and cations and low molecular weight molecules, including glycolytic intermediates, vitamins, amino acids, nucleotides, as well as some of the more important second messengers involved in cell signaling (Niessen et al., 2000; Beltramello et al., 2005; Bedner et al., 2006; Hernandez et al., 2007; Kanaporis et al., 2008).

Unpaired connexin hemichannels in the cell plasma membrane are kept in a predominantly closed state by at least one of the following mechanisms (Saez et al., 2005; Fasciani et al., 2013; Saez and Leybaert, 2014): (a) pore occlusion by mM levels of extracellular Ca^2+^ and Mg^2+^; (b) negative membrane potential; (c) post-translational modifications (e.g., phosphorylation). Open hemichannels lack ion selectivity and mediate autocrine/paracrine signaling by the release or uptake of small molecules including ATP, amino acids, reduced glutathione, NAD+, prostaglandin E_2_, and cyclic nucleotides, which are critical for cell-cell communication and the regulation of inflammatory responses (Saez and Leybaert, 2014).

Mutations leading to changes in properties, regulation or expression of connexin-made channels have been implicated in 28 distinct human hereditary diseases (Srinivas et al., 2017). Eight of these, including KID/HID syndrome, result from *GJB2* gene variants. Four diseases, including clouston syndrome or hidrotic ectodermal dysplasia (HED), have been linked to variants of *GJB6*, a gene located just 30 kb telomeric to *GJB2* on chromosome 13. These two genes encode respectively human connexin 26 (Cx26, where 26 indicated the molecular weight of the protein in kDa) and connexin 30 (Cx30), two structurally and functionally related gap junction proteins expressed in skin and inner ear (Forge et al., 2003; Zhao and Yu, 2006). Epidermal disorders associated with pathological variants of Cx26 or Cx30, for which there is no cure, can be highly incapacitating or disfiguring, and in some cases even fatal (Martin and van Steensel, 2015).

Mouse models for Cx26 and Cx30 mutants displayed auditory (Leibovici et al., 2008; Wingard and Zhao, 2015) and skin phenotypes of variable severity (Mese et al., 2011; Schutz et al., 2011; Bosen et al., 2014, 2015; Garcia et al., 2016). Of the thirteen dominant Cx26 mutations responsible for KID/HID syndrome (G11E, G12R, N14K, N14Y, S17F, I30N, A40V, G45E, D50A, D50N, D50Y, A88V) at least eight are thought to translate into hyperactive or “leaky” hemichannels (G11E, G12R, N14K, N14Y, A40V, G45E, D50A, D50N, A88V) (Retamal et al., 2015) that do not close correctly in response to extracellular Ca^2+^ or show an altered permeability to this critically important cation and second messenger (Stong et al., 2006; Gerido et al., 2007; Lee and White, 2009; Mhaske et al., 2013; Garcia et al., 2015) compromising cell viability *in vitro* (Stong et al., 2006; Lee and White, 2009). Of note, mice with ectopic expression of wild type Cx26 from the epidermis-specific involucrin promoter demonstrated increased ATP release in keratinocytes, which delayed epidermal barrier recovery and promoted a psoriasiform response (Djalilian et al., 2006). Therefore, it has been proposed that increased ATP levels act as a paracrine messenger and, by altering epidermal factors that control the proliferation and differentiation of keratinocytes, play a critical role in the etiology of skin conditions (Essenfelder et al., 2004; Garcia et al., 2016). Lack of non-toxic inhibitors with defined mechanisms of action poses a serious obstacle to therapeutic interventions for diseases caused by aberrant connexin hemichannels (Saez and Leybaert, 2014; Bruzzone, 2015).

Antibodies have become a key tool in drug discovery. Their ability to bind an antigen with a high degree of affinity and specificity is leading them to become the largest and fastest growing class of therapeutic proteins (Frenzel et al., 2016). Here, we report the identification of a human recombinant antibody that perturbs hemichannel function. For antibody discovery, we screened a single-chain fragment variable (scFv) combinatorial antibody library expressed in phage (Zhang et al., 2012). Each scFv of human immunoglobulins is formed by a heavy chain variable domain, V_H_, connected by a linker to a light chain variable domain, V_L_ (Lerner, 2016). The advantage of a synthetic phage library (McCafferty et al., 1990; Barbas et al., 1991; Breitling et al., 1991; Burton et al., 1991; Clackson et al., 1991) is that it enables full control over the *in vitro* selection process, allows to obtain highly functional binders rapidly and at lower cost, does not rely on adjuvants which can unfold proteins, and may contain over 40 billion highly stable human antibody clones (Weber et al., 2014). The antibody we selected is not toxic to cells, reversibly inhibits hemichannel currents and strongly reduces the activity of some Cx26 hyperactive mutants.

## Materials and methods

### Statistical analysis

Data are given as mean ± standard error of the mean (s.e.m.). Comparisons between two or more groups were conducted by using two-tailed analysis of variance (ANOVA) or paired *t*-test, as indicated in the figure legends. The Mann–Whitney U-test was used for data which were not normally distributed and/or had dissimilar variance. *P*-values are indicated by letter *P*, where *P* < 0.05 was selected as the criterion for statistical significance. No statistical methods were used to predetermine sample size. The experiments were not randomized and the investigators were not blinded to allocation during experiments and outcome assessment. No pre-established criteria were used to include/exclude samples or animals.

### Cell lines

All cell lines were routinely checked for mycoplasma contamination using the Venor® GeM Classic kit (Minerva Biolobs, Cat. No. 11-1025).

The FreeStyle™ 293-F cell line (ThermoFisher, Cat. No. R79007) was maintained in Freestyle 293 Expression Medium (ThermoFisher, Cat. No. 12338026). The XL1-Blue cell line (Agilent, Cat. No. 200228) was maintained in SB medium composed of 32 g/L tryptone (Oxoid, Cat. No. LP0042), 20 g/L yeast extract (Oxoid, Cat. No. LP0021), 5 g/L sodium chloride (Shanghai Sangon Biotech, Cat. No. ST1218—1 kg), and 20 μg/ml tetracycline (ShanghaiSangon Biotech, Cat. No. BS731—10 ml), pH 7.5. The culture was shaken at 37°C until an optical density (OD) of 0.5–0.7 was obtained at 600 nm.

HeLa DH cells (Sigma-Aldrich, Cat. No. 96112022) or HaCaT cells (Cell Lines Service GmbH, Cat. No. 300493) were seeded onto round glass coverslips (Fisher Scientific, Cat. No. FIS#12-542A) at different degrees of confluence, depending on the type of assay. Cells were maintained in Dulbecco's modified Eagle's medium (ThermoFisher, Cat.No. 41965039) containing 10% (v/v) FBS (Gibco-Invitrogen, Cat.No. 10270-106) and 1% penicillin/streptomycin (Gibco-Invitrogen, Cat.No. 15070-063). Twenty four hours after plating, a lipofectamine transfection reagent (Lipofectamine 2000, Cat. No. 11668-019) was used to transiently transfect HeLa DH or HaCaT cells with a construct comprising the coding region of Cx26, or its G45E or D50N mutants, fused or not to Venus (a circularly permuted yellow fluorescence mutant of the green fluorescent protein) at the C-terminal end (Beltramello et al., 2005). For some experiments, HeLa DH or HaCaT cells were transduced with a lentivirus expressing Cx26, or its G45E or D50N mutants, fused to Venus, either directly or after application of doxycycline.

### Patch clamp electrical measurements

Glass coverslips with adherent connexin-expressing HeLa DH or HaCaT cells were continuously superfused at 2 ml/min at 20–23°C with an extracellular solution (EXP) containing (in mM): 140 NaCl, 5 KCl, 10 HEPES, 2 sodium pyruvate, 4 tetraethylammonium chloride (TEA-Cl), 4 CsCl, 5 glucose, and a reduced (0.2 mM) Ca^2+^ concentration (pH 7.4, 323 mOsm).

Cells expressing Cx26–Venus were selected for patch clamp recordings by visual inspection using fluorescence microscopy, whereas cells expressing untagged Cx26 were selected based on their ability to uptake Lucifer Yellow (CH dilithium salt, Sigma-Aldrich, Cat. No. L0259) (DeVries and Schwartz, 1992; Saez and Leybaert, 2014) after 5 min of incubation in Ca^2+^-free (0 mM) EXP containing 1 mM of the dye.

Patch pipettes were filled with an intracellular solution containing (in mM): 115 KAsp, 10 NaCl, 10 KCl, 1 MgCl_2_, 10 HEPES, 1 CaCl_2_, and 5 BAPTA tetrapotassium salt (pH 7.2, 311 mOsm) and filtered through 0.22-mm pores (Millipore). Filled pipettes had resistances of 4–6 MΩ when immersed in EXP. Hemichannel currents were assayed in EXP under whole cell patch clamp recording conditions.

The antibody was dissolved in EXP and delivered through a glass micropipette pulled to a 8 μm diameter tip from B150F glass (World Precision Instruments, Sarasota, USA). During antibody delivery, the superfusion was stopped. To block connexin hemichannels, CaCl_2_ was added at the indicated final concentrations to EXP, whereas ZnCl_2_ was added at 100 μM final concentration.

### ATP release assay

HaCaT cells were transduced with a doxyxycline-inducible lentivirus expressing Cx26 and plated at a density of 1.5 × 10^3^ cells/well in 96-well plates 24 h before the experiment. Cx26 expression was induced by doxycycline addition to the culture medium 8 h before the measurement of ATP release. Complete DMEM was removed and cells were washed once with serum-free DMEM. Plates were then incubated for 30 min at 37°C and 5% CO_2_. In order to test the ability of the antibody to inhibit ATP release through Cx26 hemichannels, abEC1.1 was subsequently added to the wells to a final concentration of 400 nM, and cells were incubated in the above-mentioned conditions for an additional 30 min. Immediately before starting the ATP release-stimulation protocol, cells were washed once with a solution (NCS) containing a normal (1.8 mM) Ca^2+^ concentration and (in mM): 137 NaCl, 5.36 KCl, 0.81 MgSO_4_, 0.44 KH_2_PO_4_, 0.18 Na_2_HPO_4_, 25 HEPES, and 5.55 Glucose (pH7.3). Cells were then washed a second time either with the same NCS or with a solution (ZCS) containing 0 mM Ca^2+^ concentration and (in mM): 137 NaCl, 5.36 KCl, 0.44 KH_2_PO_4_, 0.18 Na_2_HPO_4_, 0.1 EGTA, 25 HEPES, and 5.55 Glucose (pH7.3). To stimulate the release of ATP through Cx26 hemichannels, cells were incubated in NCS, ZCS, or ZCS supplemented with abEC1.1 (400 nM) for 10 min at 37°C. The amount of ATP released in these conditions was measured with a bioluminescent ATP assay kit (Molecular Probes-A22066) and bioluminescence was measured by a luminometer (Perkin Elmer Victor Light 1420). For each experiment, ATP standard curves were generated using serially-diluted concentrations of ATP and were used to convert measurements of luminescence signals into ATP concentrations.

### Lactate dehydrogenase (LDH) release assay

LDH release was estimated by measuring optical absorbance (*A*) at 490 nm, using a Cytotoxicity LDH Assay Kit-WST (Dojindo Molecular Technologies, Cat. No. CK12) according to the manufacturer's instruction. Briefly, HaCaT cells were seeded in 96-well plates at 8,000 cells per well density and cultured in 100 μl DMEM medium (HyClone, Cat. No. SH30022.01) without sodium pyruvate and penicillin/streptomycin antibiotics, and supplemented with 5% FBS (Gibco, Cat. No. 10099141). For each sample, *A* was measured by a PerkinElmer Enspire plate reader. Untransfected cells incubated in normal medium were used as minimum absorbance (*A*_*min*_) controls, whereas untransfected cells exposed to 1% Triton X-100 for 30 min before testing were used as maximum absorbance (*A*_*max*_) controls. Percent LDH release (*R*) was estimated by substituting *A*-values into the following formula:

$$\begin{array}{l} R=\frac{A-A_{min}}{A_{max}-A_{min}}\times 100 \end{array}$$

and averaging the results over homogenous sets of eight wells.

### Cochlear organotypic cultures

#### Ethics statement

All experiments involving the use of animals (mice) were performed in accordance with a protocol approved by the Italian Ministry of Health (Prot. n.1276, date 19/01/2016).

#### Preparation

Cochleae from P5 C57BL6/N mice of both sexes were quickly dissected in ice-cold Hepes buffered (pH 7.2) HBSS (ThermoFisher, Cat. No. 14025050), placed onto glass coverslips coated with Cell-Tak (Biocoat, Cat.No. 354240) and incubated overnight at 37°C in DMEM/F12 (ThermoFisher, Cat. No. 11320-074) supplemented with 5% FBS (ThermoFisher, Cat. No. 10270-106) and 100 μg/ml ampicillin (Sigma-Aldrich, Cat.N. A0166).

#### Ca^2+^ imaging and dye transfer assays

Cochlear cultures were incubated for 40 min at 37°C in DMEM/F12, supplemented with fluo-4 AM (16 μM, Thermo Fisher Scientific, Cat. No.F-14201). The incubation medium contained also pluronic F-127 (0.1%, w/v), and sulfinpyrazone (250 μM) to prevent dye sequestration and secretion. Samples were then transferred on the stage of a spinning disk confocal microscope (DSU, Olympus) and perfused with extracellular solution for 20 min at 1 ml/min to allow for de-esterification. The perfusion medium (EXM) contained (in mM): NaCl 135, KCl 5.8, CaCl_2_ 1.3, NaH_2_PO_4_ 0.7, MgCl_2_ 0.9, Hepes-NaOH 10, d-glucose 6, pyruvate 2, amino acids, and vitamins (pH 7.48, 307 mOsm). The abEC1.1 antibody was diluted in EXM at a final concentration of 952 nM and applied to the cochlear preparation for 20 min before starting the recording. The antibody was delivered through a glass micropipette pulled to a 8 μm diameter tip on a vertical two-stage puller (PP-830, Narishige) from B150F glass (World Precision Instruments, Sarasota, USA). Experiments were performed at near-physiological temperature (34–36°C).

Fluorescence excitation was produced by light emitted by a 488 nm diode laser (COMPACT-150G-488-SM, World Star Tech) filtered through a narrow band filter (Semrock, Cat. No. FF02-482/18-25). Fluo-4 emission was filtered through a 535/43 m OD6 bandpass interference filter (Edmund Optics). Confocal fluorescence images were formed by a water immersion objective (40x, N.A. 0.8, LumPlanFL, Olympus) and projected on a scientific-grade camera (PCO.Edge, PCO AG) controlled by software developed in the laboratory. Images were acquired at one frame per second with a typical exposure time of 100 ms, stored on disk and processed off-line using the Matlab 7.0 software package (The MathWorks Inc.). Ca^2+^ signals were quantified pixel-by-pixel as relative changes of fluorescence emission intensity (Δ*F*/*F*_0_), where *F*_0_ is fluorescence at the beginning of the recording, *F* is fluorescence at time *t* and Δ *F* = *F* −*F*_0_.

For dye transfer assays, patch clamp glass micropipettes were filled with Lucifer Yellow, which is broadly used also as gap junction channel permeability probe (Hernandez et al., 2007), dissolved at 220 μM (final concentration) in a 320 mOsm intracellular solution containing (in mM): KCl 134, NaCl 4, MgCl_2_ 1, HEPES 20, EGTA 10 (adjusted to pH 7.3 with KOH), and filtered through 0.22 μm pores (Millipore). Pipette resistances were 3–4 MOhm when immersed in the bath. One cell (donor) was patch clamped and maintained in the cell-attach configuration for a few seconds to establish a baseline. The patch of membrane under the pipette sealed to the donor cell was subsequently ruptured, allowing Lucifer Yellow to fill the cell, while leaving the seal intact (whole cell recording conditions). The cell was held at its zero current level using the current clamp configuration of the patch clamp amplifier (Axopatch 200B, Molecular devices). Fluorescence images were acquired at one frame per second with a typical exposure time of 100 ms, and displayed as (*F* – *F*_bck_)/(*F*_max_ – *F*_bck_), where *F*_max_ is the asymptotic maximal value reached in the injected cell and *F*_bck_ is autofluorescence.

### Selection of Cx26 antibodies from combinatorial antibody phage libraries

A peptide corresponding to residues 41–56 of Cx26 (KEVWGDEQADFVCNTL = pepEC1.1) was synthesized and labeled with biotin at the N-terminus (Chinese Peptide Company, Hangzhou Economic and Technological Development Zone, China).

Streptavidin-coated magnetic beads (Pierce, Cat. No. 88817) were mixed in Tween phosphate buffer (PBST) with biotinilated pepEC1.1 and screened against a scFv combinatorial library expressed in phage (Zhang et al., 2012). For the phage panning process (Barbas et al., 2004; Lee et al., 2007), specific clones were enriched by binding to immobilized pepEC1.1, followed by elution with Glycine-HCl (pH 2.2) and repropagation of phage in XL1-Blue cells. After four rounds of panning, 150 colonies were picked and analyzed by phage ELISA (see below). Positive colonies were sequenced and analyzed using the international ImMunoGeneTics information system® (Lefranc et al., 2015). Complementarity determining regions 3 (CDR3s) were aligned with Clustal X (Larkin et al., 2007) and a phylogenetic tree was constructed using CLC Genomics Workbench 8.0 (CLCbio). The genes encoding the scFv candidates, identified by analysis of the phylogenetic tree, were cloned into a modified pFUSE expression vector (Invivogen, Cat. No. pfuse-hg1fc2) to obtain scFv-Fc fusion proteins. 293-F cells were transfected with the scFv-Fc vectors and the expressed fusion proteins were purified using HiTrap Protein A HP columns (GE Healthcare, Cat. No.17-0403-03) with ÄKTApurifier 100 (GE Healthcare). After purification, the buffer was exchanged to PBS (pH 7.4). The purified scFv-Fc proteins were kept either at 4°C, for short-term storage, or at −80°C for longer-term conservation.

### Western blot analyses

The Cx26 coding sequence fused with 10^*^His and flag tag was cloned into a pFastBac1 baculovirus transfer vector and expressed using the Bac-to-Bac® Baculovirus Expression System (Life Technologies, Cat. No. 10359-016, 10360-014, 10584-027, 10712-024). The baculovirus infected Sf9 cells were cultivated in an ESF 921 insect cell culture medium (Expression Systems, Cat. No. 96-001-01) at 27°C for 48 h. The recombinant Cx26 protein was then purified using a modified protocol described previously (Dupont et al., 1991; Stauffer et al., 1991).

Alternatively, Hela DH cells transfectants expressing Cx26-Venus were used to isolate total cellular protein. Around 0.1 μg of protein supernatant was separated in a denaturating 10% polyacrylamide gel and transferred to a nitrocellulose membrane by electroblotting.

After blocking with 5% milk powder in washing buffer (TBST, composed of 10 mM Tris-HCl, 150 mM NaCl, 0.1% Tween-20, pH 7.5) for 1 h at room temperature, the nitrocellulose membrane was incubated overnight at 4°C with scFv-Fc fusion proteins (1 mg/ml, dilution factor 1:100). Membranes were washed three times with TBST and after 1 h they were incubated with goat anti-human horseradish peroxidase (HRP)-conjugated antibody (dilution factor 1:2,000; Sigma, Cat. No.A0170), which recognizes the Fc domain of the scFv-Fc fusion proteins. Finally, membranes were incubated with SuperSignal West Picochemiluminescence substrate (Pierce, Cat. No. 34087), which reacts with HRP, and examined using Bio-Rad ChemiDoc MP (Biorad).

### ELISA and phage elisa

Ninety-six-well ELISA plates (Corning Costar, Cat. No. 3690) were filled with 25 μl per well of a solution containing avidin (1 μg/well; Pierce, Cat. No. 21121) dissolved in CBS buffer (pH 9.0). Plates were incubated at 4°C overnight or 37°C for 1 h. After washing with PBST buffer (0.05% TWEEN20 in PBS), 25 μl of a pepEC1.1 solution (0.05 μg/well, diluted in PBS) was added to each well and incubated for 1 h at 37°C. Wells were washed twice and blocked with 150 μl per well of M-PBST (5% milk in PBST) and incubated for 1 h at 37°C. After discarding the blocking solution, 25 μl of abEC1.1 solution (1 mg/ml, diluted in M-PBST buffer, dilution factor 1:100) were added and incubated for 1 h at 37°C, then washed eight times. Each well then received 50 μl of a solution containing goat anti-Human horseradish peroxidase-conjugated antibody (Sigma, Cat. No. A0170-1 ml) diluted in M-PBST buffer (dilution factor 1:5,000). Plates were incubated for 1 h at 37°C and then washed eight times. Finally, 50 μl of substrate ABTS solution (Roche, Cat. No. 11684302001) were added to each well and incubated for 20 min at room temperature. OD was read out at 405 nm on a PerkinElmer Enspire plate reader.

Relative affinity and specificity of scFv-phages and soluble scFvs was assessed against the pepEC1.1 antigen. To this end, 10 μg/ml of pepEC1.1 were used to coat a microtiter plate at 4°C overnight. Any remaining binding sites were blocked with Blotto (5% w/v of Bovine Serum Albumin in PBST; bovine serum albumin was purchased from Thermo Fisher, Cat. No. 23210). Approximately 25 μl per well of scFv-phage or soluble scFv supernatant from overnight cell cultures was added and incubated for 1 h at 37°C. For scFv-phage ELISA (Zhang et al., 2012), after washing, 25 μl of anti-M13 mAb horseradish peroxidase (HRP) conjugate (GE Healthcare, Cat. No. 27-9421-01) diluted 1:1000 in Blotto was added for 30 min at 37°C. For ELISA using soluble scFv, anti-Human horseradish peroxidase conjugate (Sigma, Cat. No. A0170-1 ml) in Blotto was added and incubated for 30 min at 37°C. Detection was accomplished by adding 50 μl of ABTS solution (Roche, Cat. No. 11684302001) and absorbance was measured at 405 nm.

### Antibody crystallization, diffraction data collection, structure determination, and refinement

Crystallization was carried out using the hanging drop vapor diffusion method. Crystals of the scFv domain of abEC1.1 grew in the drop consisting of 0.2 μL of the protein solution (8 mg/mL) and 0.2 μL of the reservoir solution (0.1 M HEPES, pH7.5, 3 M NaCl). Crystals were cryoprotected using the reservoir solution supplemented with 20% v/v glycerol and then flash cooled into the liquid nitrogen. Diffraction data were collected at the BL18U beam line of Shanghai Synchrotron Radiation Facility and processed with the HKL-3000 suite (Minor et al., 2006). The structure of the scFv domain of abEC1.1 was solved with the MR method using the programs Phaser (McCoy, 2007). The Fv fragment of a human antibody structure (PDB ID 2GHW) was used as the search model. Structure refinement was carried out using Refmac5 (Murshudov et al., 2011) and model building was facilitated with the program Coot (Emsley and Cowtan, 2004).

### Molecular modeling and dynamics

The atomistic model of V_H_ and V_L_ (without linker) derived from the crystal structure was docked to the extracellular domain of a published model of Cx26 hemichannel embedded in the plasma membrane (Zonta et al., 2012) using the ClusPro 2.0 server (Comeau et al., 2004) in the antibody docking mode (Brenke et al., 2012). Among the 50 docking configuration generated by the software, we selected the only one in which the three CDRs of V_H_ faced the EC1 loop of Cx26, and we tested its stability by performing a 70 ns molecular dynamics simulation using the Gromacs 4 package (Pronk et al., 2013) and the CHARMM 36 force field (Best et al., 2012). After molecular dynamics thermalization, we obtained a stable configuration in which V_H_ and V_L_ interacted with three adjacent protomers of the Cx26 hemichannel. Therefore, we added a second symmetrically docked pair of V_H_ and V_L_ and obtained a new configuration in which the pore lumen was completely covered.

The atomistic model system, containing the Cx26 hemichannel inserted in a phospholipid bilayer and the pair of docked antibodies, underwent a short energy minimization in vacuum, and was subsequently solvated with full atom TIP3P water, containing Cl^−^ and K^+^ ions at a concentration of ~0.15 M in order to mimic a physiological ionic strength. After solvation, the total number of atoms was around 253,000. We then performed an equilibrium molecular dynamics simulation under periodic boundary conditions at constant pressure for an additional 200 ns.

Temperature *T* was kept fixed at 300 K in all simulations, and, where stated, the pressure P was fixed at 1 atm using Berendsen thermostat and barostat (Berendsen et al., 1984). Fast smooth Particle-Mesh Ewald summation (Darden et al., 1993) was used for long-range electrostatic interactions, with a cut off of 1.0 nm for the direct interactions.

## Results

### Antibody selection

Based on the crystal structure of Cx26 gap junction channels (Maeda et al., 2009) and a related molecular dynamics model of the Cx26 hemichannel (Zonta et al., 2012), we synthesized a bait peptide, which we named pepEC1.1, corresponding to residues 41–56 in the EC1 domain of Cx26. We investigated the conformation of pepEC1.1 in solution by circular dichroism (CD) and nuclear magnetic resonance (NMR) spectroscopy, and determined that the peptide did not fold in solution, therefore we screened the scFv phage library (Zhang et al., 2012) against pepEC1.1.

**Figure 1:**
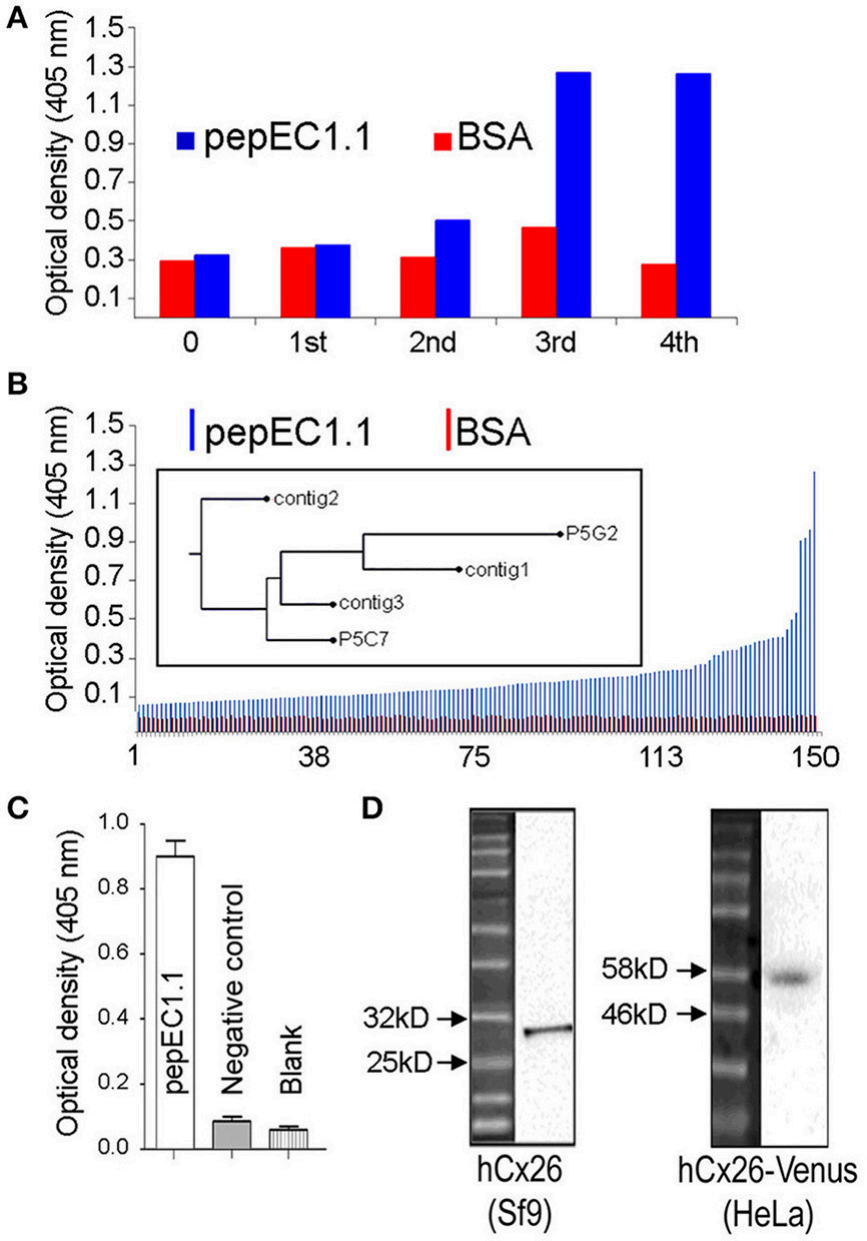
Selection of antibodies in phage. **(A)** Optical density readout from a plate reader vs. panning round for a negative control antigen (BSA, red) and the pepEC1.1 bait peptide (blue); only pepEC1.1 shows a signal increase after two rounds of enrichment. **(B)** Phage ELISA results showing optical density readout from the plate reader vs. colony number, ranked by increasing signal intensity. Positive clones (pepEC1.1/BSA ratio > 2) were sequenced and a phylogenetic tree based on the heavy-chain CDR3 sequences was constructed (inset; clones are labeled by the protocol used to obtain them and the clone number of each protocol). **(C,D)** After purification, the binding specificity of the candidate antibody, abEC1.1, was tested by ELISA, against pepEC1.1 **(C)**, and by western blot, against Cx26 proteins **(D)**, fused with a 10^*^His and flag tag (29.4 kDa) and purified from a Sf9 expression system (left), or from proteins obtained from HeLa DH transfectants expressing the 52 kDa chimeric protein Cx26-Venus (right). The negative control peptide in **(C)** corresponds to residues 172 to 184 of Cx26 (sequence: AWPCPNTVDCFVSR), which form part of EC2.

After two rounds of panning (see section Materials and Methods), an increased enzyme-linked immunosorbent assay (ELISA) signal was observed for the pepEC1.1 group compared to the control group (bovine serum albumin, BSA); the scFvs which specifically bound pepEC1.1 were enriched after four rounds of panning (Figure 1A). Seven hundred phage-infected clones were picked randomly from the third and fourth round of panning and tested for their ability to bind pepEC1.1 using phage ELISA. One hundred and fifty phage clones were selected from the original 700 clones as positive binders (pepEC1.1/control >2) (Figure 1B). Positive colonies were sequenced, complementarity-determining regions 3 (CDR3) were aligned and a phylogenetic tree was constructed (Figure 1B, inset). Based on the analysis of the phylogenetic tree, different scFv sequences were converted to the scFv-Fc format, in which the scFv domain is fused in one polypeptide chain to the hinge and fragment constant (Fc) domain of human immunoglobulin G1 (IgG1) (Bujak et al., 2014). The binding affinity of the scFv-Fc recombinant antibodies for pepEC1.1 was quantified by ELISA, and the most promising scFv-Fc candidate (Figure 1C) was named abEC1.1.

In western blot analyses of Cx26 protein fused with a 10^*^His and flag tag (29.4 kDa) and purified from a Sf9 expression system (see section Materials and Methods), abEC1.1 showed a band between 25 and 32 kDa (Figure 1D, left). Likewise, using total cellular proteins obtained from HeLa DH cells (a clone virtually devoid of native connexins) transfected with Cx26-Venus (Beltramello et al., 2005), abEC1.1 showed a band between 46 and 58 kDa (Figure 1D, right), in agreement with the expected molecular weight of Cx26-Venus (52 kDa).

### The abEC1.1 antibody inhibits connexin hemichannel currents

**Figure 2:**
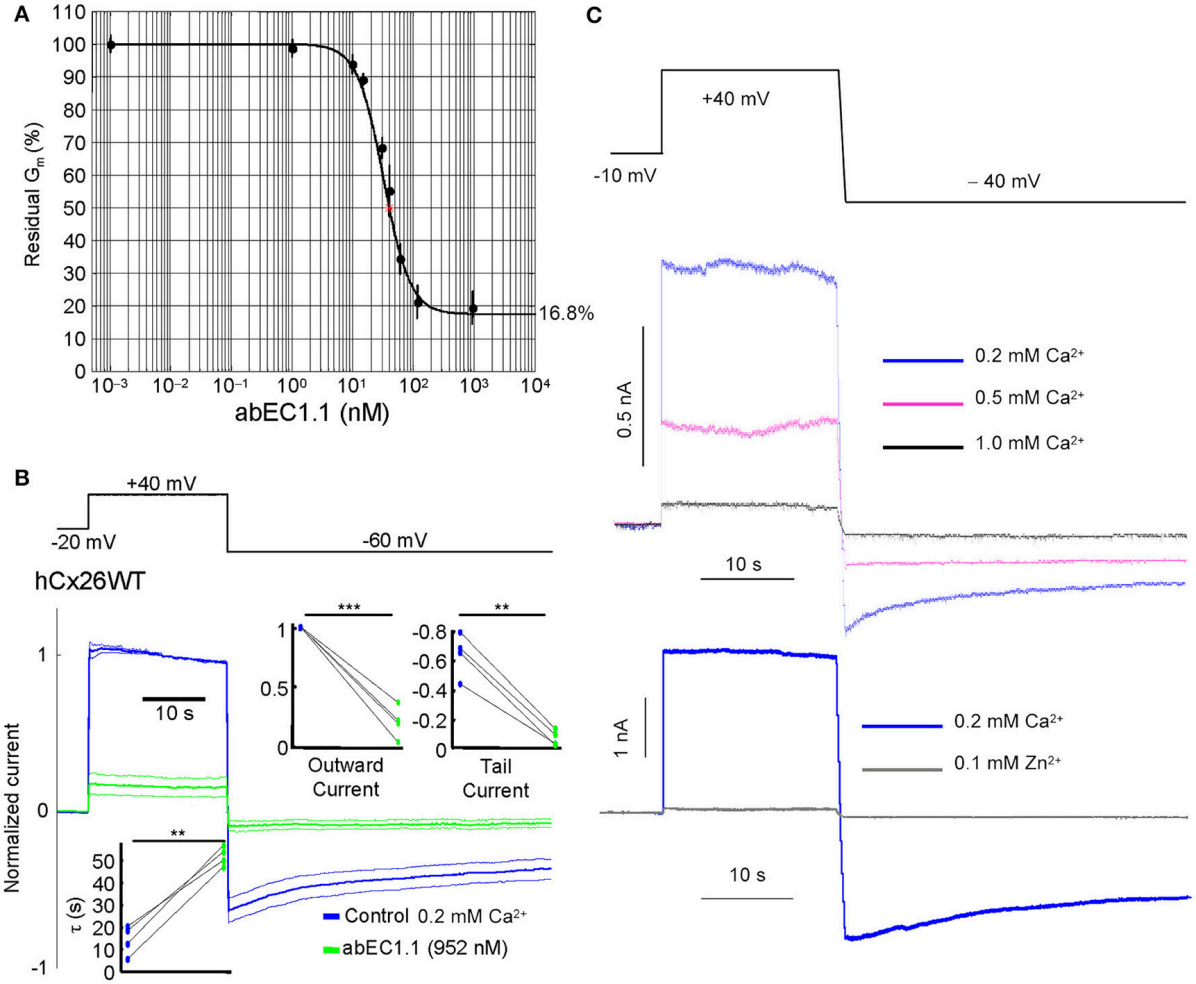
The abEC1.1 antibody inhibits Cx26 hemichannel currents with IC_50_ of ~40 nM. **(A)** Membrane conductance measured with the step protocol in **(B)** and normalized to pre-antibody application levels, vs. abEC1.1 concentration. Each data point is the mean ± s.e.m. for *n* = 5 cells; the solid line is a least-square fit with a modified Hill equation *y* = α [1+(*x*/γ)^*n*^]^−1^ + β, where *x* is antibody concentration (in nM), α = 0.832, β = (1 −α) = 0.168, γ = 36.926 nM, and *n* = 2. **(B)** Representative whole cell currents elicited by shown voltage commands (top, black trace) in HeLa DH cells transiently transfected with Cx26-Venus; shown are mean (thick traces) ± s.e.m. (thin traces) for *n* = 5 cells before (blue traces, control) and after application of abEC1.1 at 952 nM for 15 min (green traces) in 0.2 mM Ca^2+^. Data were normalized to the mean value of the control response during the application of the +40 mV depolarization step. Top insets show normalized current immediately after the voltage commands to +40 mV (outward current) and −60 mV (tail current). Bottom inset shows time constant of a single exponential fit to the tail current decay over time. Asterisks indicate significance level between control condition and antibody application (^**^P < 0.01; ^***^P < 0.001, paired *t*-test). **(C)** Whole cell currents were reduced by extracellular Ca^2+^ in a concentration-dependent fashion (magenta and black traces) and were abrogated by adding 0.1 mM Zn^2+^ (gray trace) to the extracellular medium containing 0.2 mM Ca^2+^.

To determine whether abEC1.1 binding to Cx26 interferes with hemichannel function, we performed patch clamp experiments on HeLa DH cells expressing Cx26-Venus. In low extracellular Ca^2+^ conditions (0.2 mM), which favor the opening of connexin hemichannels (Saez et al., 2005; Fasciani et al., 2013; Saez and Leybaert, 2014; Bennett et al., 2016), the dose-inhibition response curve of membrane conductance, normalized to pre-antibody application levels, vs. abEC1.1 concentration yielded a half maximal inhibitory concentration (IC_50_) of ~40 nM (Figures 2A,B). The inhibitory effect of abEC1.1 on Cx26 hemichannels was incomplete, leaving a residual conductance of 17.5% ± 4.1% (*n* = 4) at saturating concentrations (952 nM). Similar results were obtained in HeLa DH cells expressing Cx26 not fused to Venus (residual conductance at 952 nM: 15.9 ± 3.4%, *n* = 4, *P* = 0.8, ANOVA).

The currents affected by the antibody were inhibited by extracellular Ca^2+^, in a dose-dependent manner, and abrogated by Zn^2+^ (100 μM; Figure 2C), a non-specific Cx26 hemichannel blocker (Sanchez et al., 2014; Levit et al., 2015).

### abEC1.1 inhibits connexin hemichannels but not gap junction channels in mouse organotypic cochlear cultures

**Figure 3:**
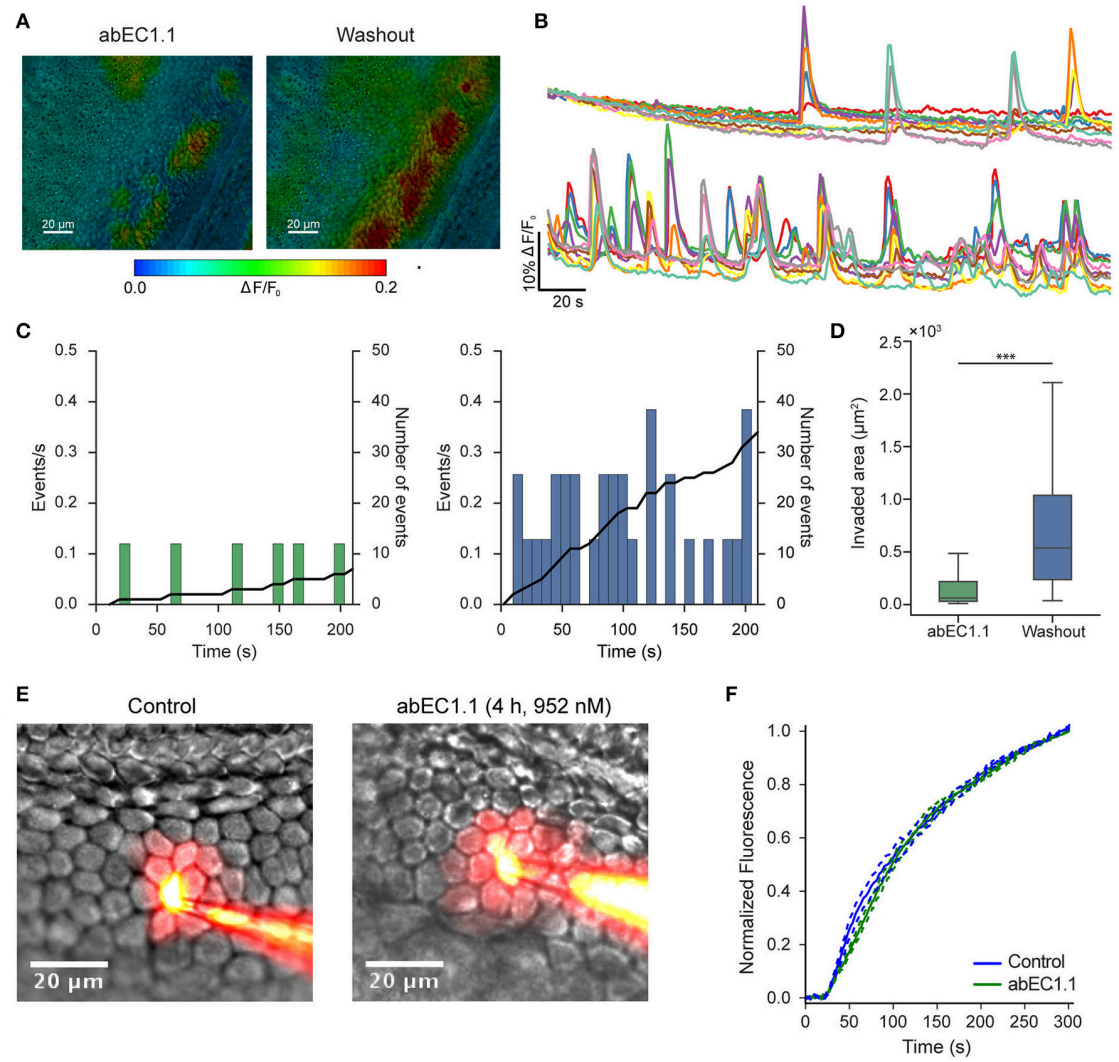
The abEC1.1 antibody inhibits connexin hemichannels but not gap junction channels in the mouse postnatal cochlea. **(A)** False-color images of fluo-4 fluorescence change (Δ*F*/*F*_0_), encoded as shown by the color scale-bar, obtained as maximal projection rendering of all frames recorded in an apical turn culture from the greater epithelial ridge of a P5 mouse imaged for 5 min at one frame per second in the presence of the abEC1.1 antibody (left) and after antibody washout with normal extracellular solution (right). The experiment was replicated two times. **(B)** Fluo-4 traces generated from the experiment in **(A)** as pixel averages from 10 non-sensory cells of the greater epithelial ridge in the presence of the antibody (952 nM, top) and after washout (bottom); **(C)** Analysis of data in **(B)**, showing frequency histograms of spontaneous cytosolic Ca^2+^ transients (events) in the presence of the antibody (952 nM, left) and after washout (right); in each graph, the solid black line (right ordinates) is the time integral of the corresponding frequency histogram and, as such, it tracks the mean number of events in the cell population from the onset of the recording. **(D)** Box plots showing distribution of areas invaded by intercellular Ca^2+^ waves in the presence of the antibody (952 nM, left) and after washout (right); invaded areas were significantly reduced in the presence of the antibody (^***^P < 0.001, two-tailed Mann-Whitney U-test). Data in **(D)** were obtained from two cochlear cultures, *n* = 117 Ca^2+^ waves in the washout and 74 in the antibody condition. Box plot conventions: the upper and lower whisker of the plot extend to the maximum value below Q3+1.5^*^IQR and the minimum value above Q1–1.5^*^IQR, respectively, where Q1 is the first quartile, Q3 is the third quartile, and IQR = Q3-Q1; outliers are not shown. **(E)** Dye transfer assays in the absence of abEC1.1 (Control, left) and after 4 h of incubation with abEC1.1 (952 nM, right). **(F)** Lucifer yellow fluorescence emission averaged over the cell body of (first order) cells adjacent to the injected cell, and normalized to the maximal fluorescence emission at *t* = 300 s; data are mean values (solid traces) ± s.e.m. (dashed lines) for *n* = 6 cells in each condition; the experiment was replicated three times.

In the mammalian cochlea, ATP is released under various conditions (Housley et al., 2009) from the organ of Corti (Wangemann, 1996; Zhao et al., 2005) and the lateral wall (Munoz et al., 2001; Chen et al., 2015). Here, we used organotypic cochlear cultures from P5 mice (Beltramello et al., 2005). In this model system, which is known to express endogenous Cx26 (Ortolano et al., 2008), the release of ATP from connexin hemichannels plays a crucial role in intercellular Ca^2+^ signaling both in the lesser epithelial ridge (Anselmi et al., 2008; Majumder et al., 2010; Ceriani et al., 2016) and in the greater epithelial ridge (Tritsch et al., 2007; Schutz et al., 2010; Rodriguez et al., 2012; Wang and Bergles, 2015; Johnson et al., 2017). Incubating cochlear organotypic cultures with abEC1.1 for 15–20 min severely but reversibly reduced the frequency of spontaneous Ca^2+^ transients in the greater epithelial ridge, as well as the area of tissue invaded by each event (Figures 3A–D and Supplementary Video 1).

Numerous studies have reported that connexins have half-lives of 1.5–3.5 h (Thomas et al., 2005; Esseltine and Laird, 2016), and that antibodies targeted to the extracellular domain of connexins may interfere with the assembly of gap junction channels (Riquelme et al., 2013). Therefore, we assayed dye transfer among non-sensory cells of the lesser epithelial ridge after incubating cochlear organotypic cultures for 4 h with abEC1.1 at a saturating concentration (952 nM). The cell-to-cell spreading of Lucifer Yellow over time was not hampered by the antibody (Figures 3E,F and Supplementary Video 2), indicating that abEC1.1 does not affect dye transfer through gap junction channels in this tissue.

**Figure 4:**
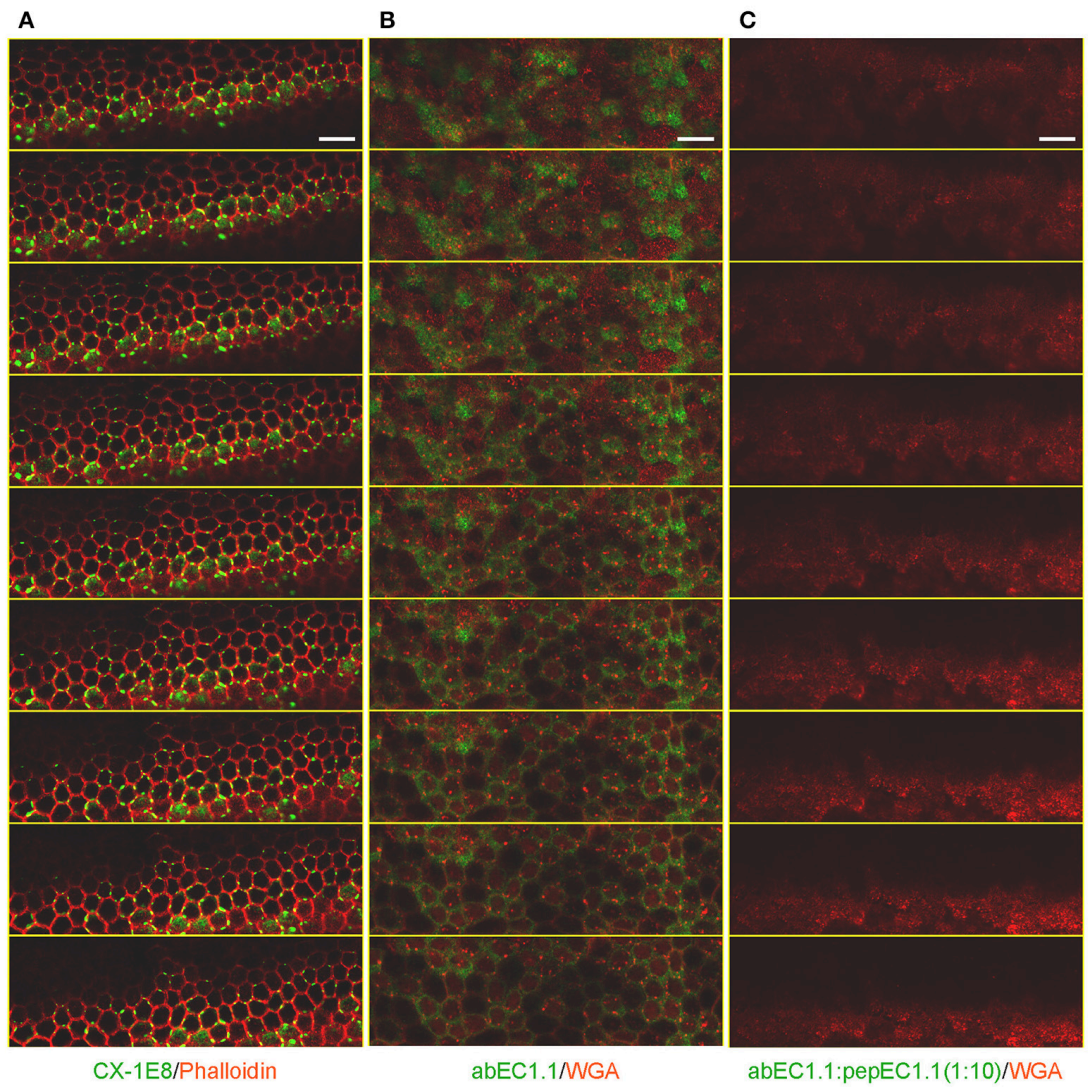
Immunolabeling of non-sensory cells in cochlear organotypic cultures from P5 C57BL6/N mice. Shown are through-focus confocal sequences (z-stacks) taken at 0.5 μm intervals under the indicated labeling conditions using a laser scanning confocal microscope (Leica, TCS SP5) equipped with a 40X objective (Leica, HCX pl apo 1.25 0.75 oil). All samples were mounted onto glass slides with a mounting medium (FluorSave™ Reagent, Merk, Cat. No. 345789). Scale bars, 25 μm. **(A)** Cultures were fixed in 4% paraformaldehyde (PFA) for 20 min at room temperature, permeabilized for 15 min with 0.1% v/v TritonX-100 at room temperature and incubated with CX-1E8, a commercial mouse monoclonal antiCx26 antibody (ThermoFisher, Cat. No. 33-5800). CX-1E8 was visualized with a goat-anti-mouse Alexa488-conjugated secondary antibody (Thermo Fisher, Cat. No. A-11029). Cells were counter-stained by labeling F-Actin with Alexa Fluor® 568 Phalloidin (ThermoFisher, Cat. No. A12380), diluted 1:200 in rinse solution, for 1 h at room temperature. **(B)** To label the plasma membrane, live cultures were counter stained with Wheat Germ Agglutinin (WGA) Texas Red®-X Conjugate (Thermofisher, Cat. No. W21405) following the manufacture's instruction. Cultures where then fixed, but not permeabilized, in 4% paraformaldehyde (PFA) for 20 min at room temperature, washed times times in PBS, then rinsed in PBS containing 2% BSA and incubated overnight at 4°C with abEC1.1 (2 μg/ml) dissolved in rinse solution (1% BSA in PBS), and finally washed three times in PBS (5 min each time). abEC1.1 was visualized with a Fluorescein AffiniPure Donkey Anti-Human IgG (H+L) secondary antibody (Jackson ImmunoResearch, Cat. No. 709-545-149), diluted 1:100 in rinse solution, and applied for 1 h at room temperature. **(C)** Cultures were incubated with abEC1.1 pre-empted by overnight incubation with cognate peptide (pepEC1.1, dissolved in DMSO) in a 1:10 (w/w) proportion and processed as in **(B)**.

The presence of gap junctions in these dye coupled cells was confirmed by immunolabeling detergent-permeabilized cochlear organotypic cultures with a commercial monoclonal anti-Cx26 antibody (Figure 4A). In non-permeabilized cultures, we detected abEC1.1 immunoreactivity in the apical plasma membrane (Figure 4B), consistent with the capacity of abEC1.1 to recognize surface-exposed Cx26 but not gap junction channels. These results are similar to those previously obtained with CELAb polyclonal antibodies (Clair et al., 2008) in organotypic cochlear cultures (Anselmi et al., 2008; Majumder et al., 2010). abEC1.1 immunoreactivity was eliminated by pre-incubating the antibody with its cognate peptide, pepEC1.1 (Figure 4C).

**Figure 5:**
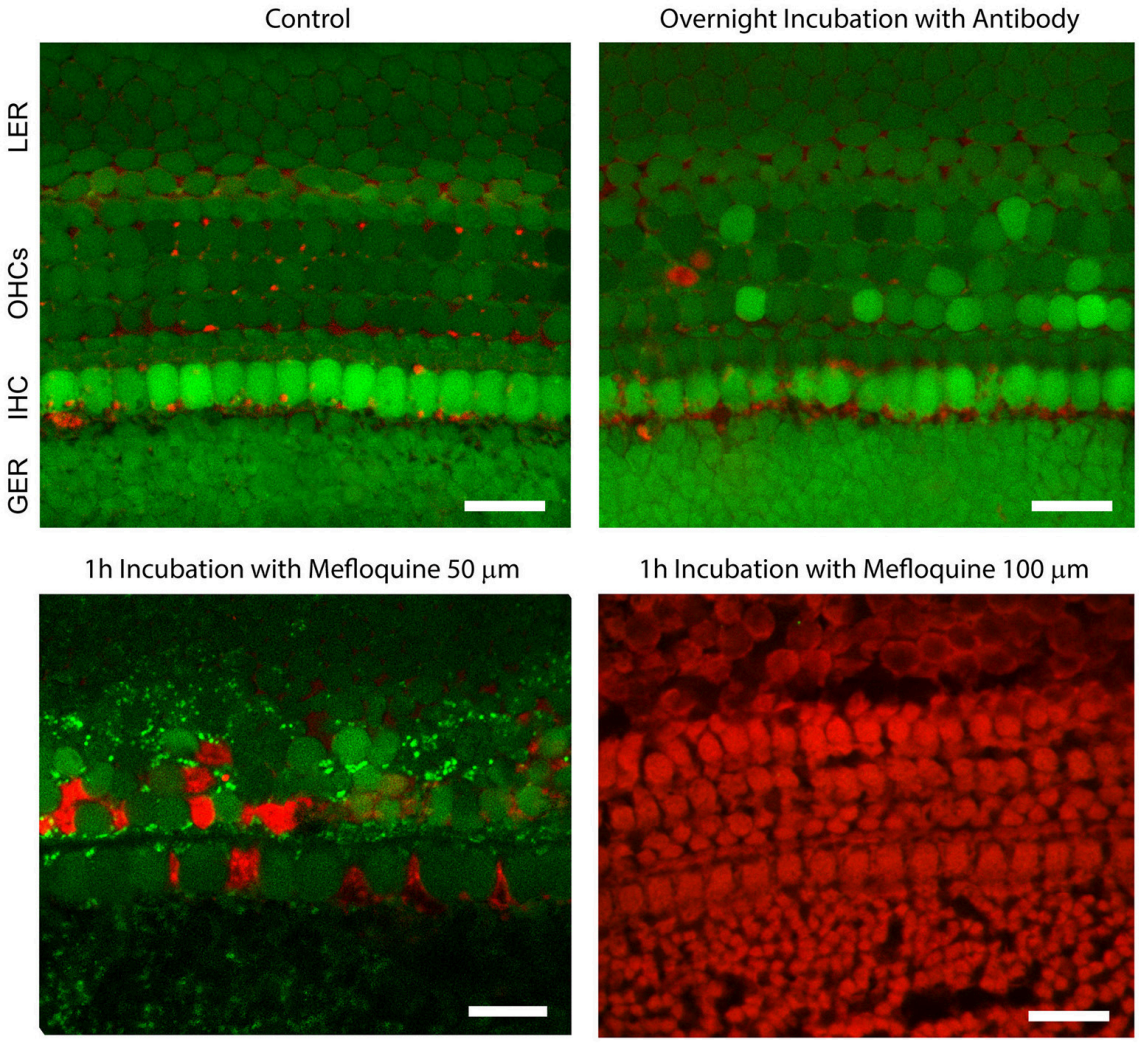
The abEC1.1 antibody is not toxic to cells of the mouse postnatal cochlea. Green, calcein AM; red, Dextran-Texas Red 70,000 mW. GER, greater epithelial ridge; IHC, inner hair cells (single row); OHCs, outer hair cells (three rows); LER, lesser epithelial ridge. Scale bars: 20 μm. For these experiments, cochlear organotypic cultures, obtained from P5 C57BL6/N mouse pups, were incubated overnight for 16 h at 37°C and 5% CO_2_ in DMEM/F12 (without FBS) supplemented with abEC1.1 (952 nM). The following day, cultures were incubated for 5 min at room temperature in DMEM/F12, supplemented with Calcein AM (5 μM, ThermoFisher, Cat. No. C1430). The incubation medium contained also 0.1% w/v pluronic F-127 and sulfinpyrazone (250 μM) to prevent dye sequestration and secretion. Cultures were then transferred to the stage of a laser scanning multiphoton confocal microscope (Bergamo 2, Thorlabs) and incubated in extracellular solution (EXM) supplemented with Dextran-Texas Red 70,000 mW (1.78 μM, ThermoFisher, Cat. No. D1830) for 30 min. Calcein fluorescence was excited at λ = 780 nm and detected with a Semrock 525/40 nm band bandpass filter (FF02-525/40-25). Texas Red fluorescence was excited at λ = 1100 nm and detected with a Semrock 625/90 nm BrightLine® CARS bandpass emission filter (FF01-625/90-25). In live cells, the non-fluorescent calcein AM is converted to a green-fluorescent dye after acetoxymethyl ester hydrolisys by intracellular estares. Labeled dextrans are membrane-impermeable polysaccharides that are excluded from the cytoplasm of live cells, but bind extracellular structures or intracellular components of cells with compromised plasma membrane.

To examine potential cytotoxic effects of the antibody, cochlear organotypic cultures were incubated overnight in serum-free culture medium supplemented with abEC1.1 (952 nM). A subsequent live/dead fluorescence assay (Figure 5) showed an overwhelming proportion of viable cells, indistinguishable from controls not exposed to the antibody. In contrast, cell viability in these organotypic cultures was compromised after just 1 h of incubation with mefluoquine, a small-molecule analog of the anti-malarian drug quinine, which requires a concentration of 100 μM (used in these assays) to inhibit Cx26 hemichannels by ~70% (Levit et al., 2015), i.e., less than the maximal inhibition imparted by abEC1.1 at (non-toxic) concentrations <1 μM.

### abEC1.1 inhibits connexin hemichannels in cultured human keratinocytes

**Figure 6:**
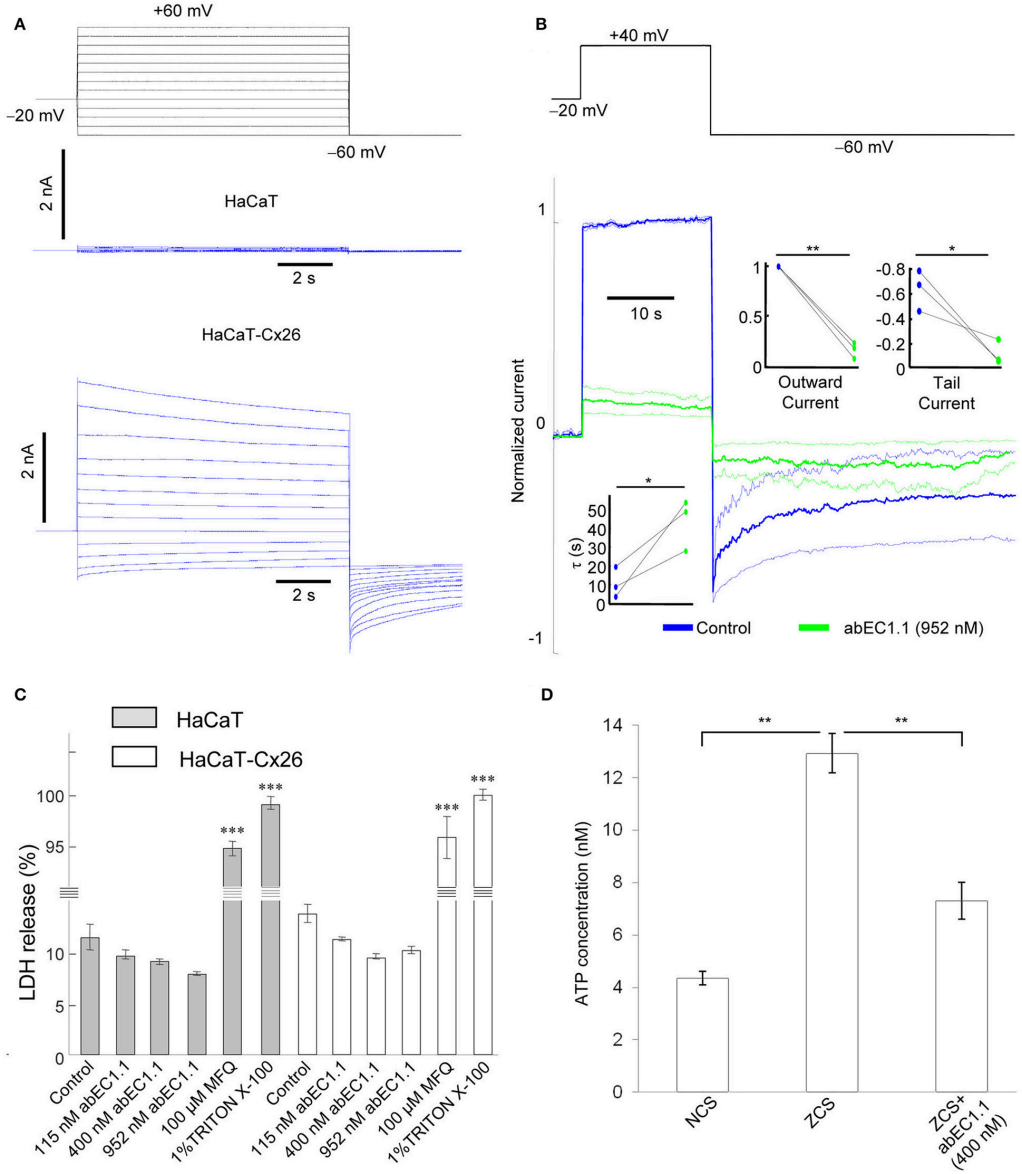
abEC1.1 inhibits keratinocytes hemichannel currents and ATP release, and is not toxic to cells. **(A)** Black traces at the top represent voltage commands applied under patch clamp conditions to elicit whole cell currents in wild type HaCaT cells (blue traces, middle) and in cells over-expressing Cx26 following lentiviral transduction (HaCaT-Cx26, blue trace, bottom); the experiment was replicated three times in each condition. **(B)** Whole cell currents elicited by shown voltage commands (top, black trace); shown are mean (thick traces) ± s.e.m. (thin traces) for *n* = 3 HaCat-Cx26 cells before (blue traces, control) and after application of abEC1.1 at 952 nM for 15 min (green traces). Data were normalized to the mean value of the control response during the application of the +40 mV depolarization step. Top insets show normalized current immediately after the voltage commands to +40 mV (outward current) and −60 mV (tail current). Bottom inset shows time constant of a single exponential fit to the tail current decay over time. Asterisks indicate significance level between control condition and antibody application (^*^P < 0.05; ^**^P < 0.01, paired *t*-test). **(C)** LDH release assay (^***^P < 0.001, ANOVA); this experiment was replicated two times. Error bars represent s.e.m. **(D)** ATP release assay (^**^P < 0.01, ANOVA); this experiment was replicated two times. NCS, normal Ca^2+^ solution (1.8 mM Ca^2+^); ZCS, nominally Ca^2+^-free solution (0 mM Ca^2+^ and no added EGTA). Data are mean values ± s.e.m. for *n* = 4 wells in each condition.

To explore further the potential applications of this antibody, we performed patch clamp experiments in a keratinocyte cell line from adult human skin (HaCaT cells) which maintain full epidermal differentiation capacity (Boukamp et al., 1988). Wild type HaCaT cells exhibited negligible whole cell currents (<200 pA) in response to voltage commands in 0.2 mM extracellular Ca^2+^, whereas currents >2 nA were measured in HaCaT-Cx26, i.e., HaCaT cells in which stable expression of Cx26 was induced by lentiviral infection (Figure 6A). abEC1.1 (952 nM) inhibited these currents by > 80% (Figure 6B), consistent with the results obtained in HeLa DH transfectants.

To test antibody cytotoxicity in keratinocytes, we used a LDH release assay. LDH is a stable enzyme, present in all types of cells, which is normally retained in the cytoplasm, but is released into the medium in the presence of cytotoxic agents (see section Materials and Methods). abEC1.1, applied overnight at saturating concentrations (115, 400, and 952 nM) reduced slightly LDH release in both wild type and HaCaT-Cx26 cells, whereas incubation with mefluoquine (100 μM) for 1 h increased LDH release by >9-fold compared to control conditions (Figure 6C). These results indicate that abEC1.1 can be safely applied to cultured keratinocytes, whereas mefluoquine is toxic. In accord with the inhibition of ATP-dependent spontaneous Ca^2+^ signaling activity in cochlear organotypic cultures, abEC1.1 (400 nm) reduced also the release of ATP from cultured keratinocytes exposed to a Ca^2+^-free medium for 10 min (Figure 6D; see section Materials and Methods for further details).

**Figure 7:**
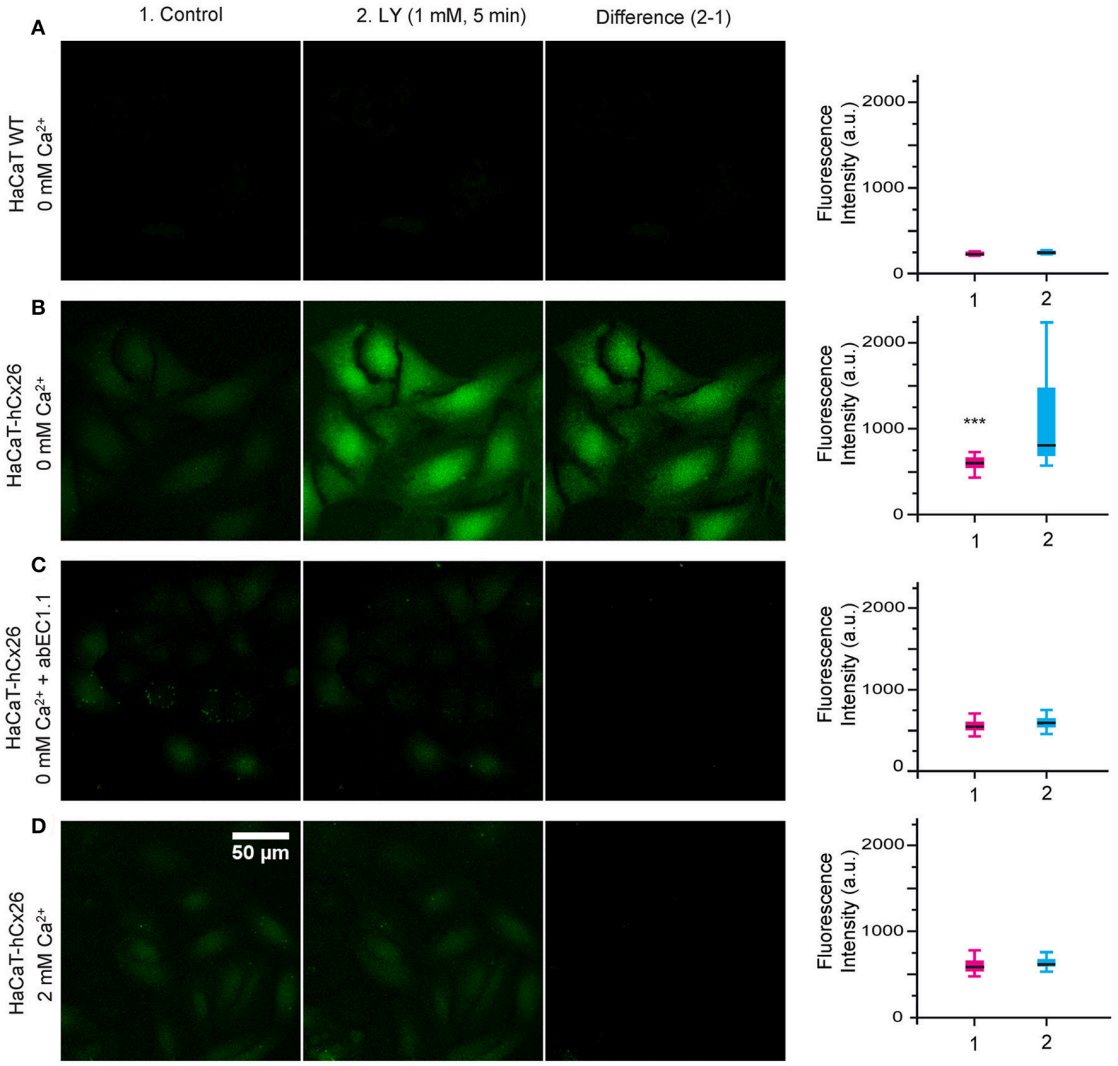
abEC1.1 inhibits dye uptake in keratinocytes. HaCaT cells, either wild type (WT, **A**) or stably expressing Cx26-Venus **(B–D)** were bathed in extracellular medium containing: **(A, B)** 0 mM Ca^2+^; **(C)** 0 mM Ca^2+^+ 952 nM abEC1.1; **(D)** 2 mM Ca^2+^. Each experiment was replicated three times. Column (1) displays the cells before incubation with Lucifer Yellow (LY); column (2) shows the same field of view after the 5 min incubation with LY (1 mM, 5 min); column (3) is the difference between the two (2-1); scale bar, 50 μm. Box plots represent the distributions of fluorescence values measured in 50 different cells before (1) and after (2) incubation with LY (^***^p < 0.001, Mann-Whitney U test).

Cellular uptake of fluorescent tracers allows real time measurements of membrane permeability properties via hemichannels (Saez and Leybaert, 2014). Lucifer Yellow uptake was undetectable in wild type HaCaT cells incubated for 5 min in nominally Ca^2+^-free (0 mM) extracellular medium (Figure 7A), but was prominent in HaCaT–Cx26 cells (Figure 7B). Dye uptake was inhibited either by pre-incubating HaCaT–Cx26 cells with abEC1.1 (952 nM) for 20 min in 0 mM Ca^2+^ (Figure 7C), or by maintaining the cells in 2 mM Ca^2+^ (Figure 7D). These results indicate that abEC1.1 abrogates dye uptake through connexin hemichannels.

**Figure 8:**
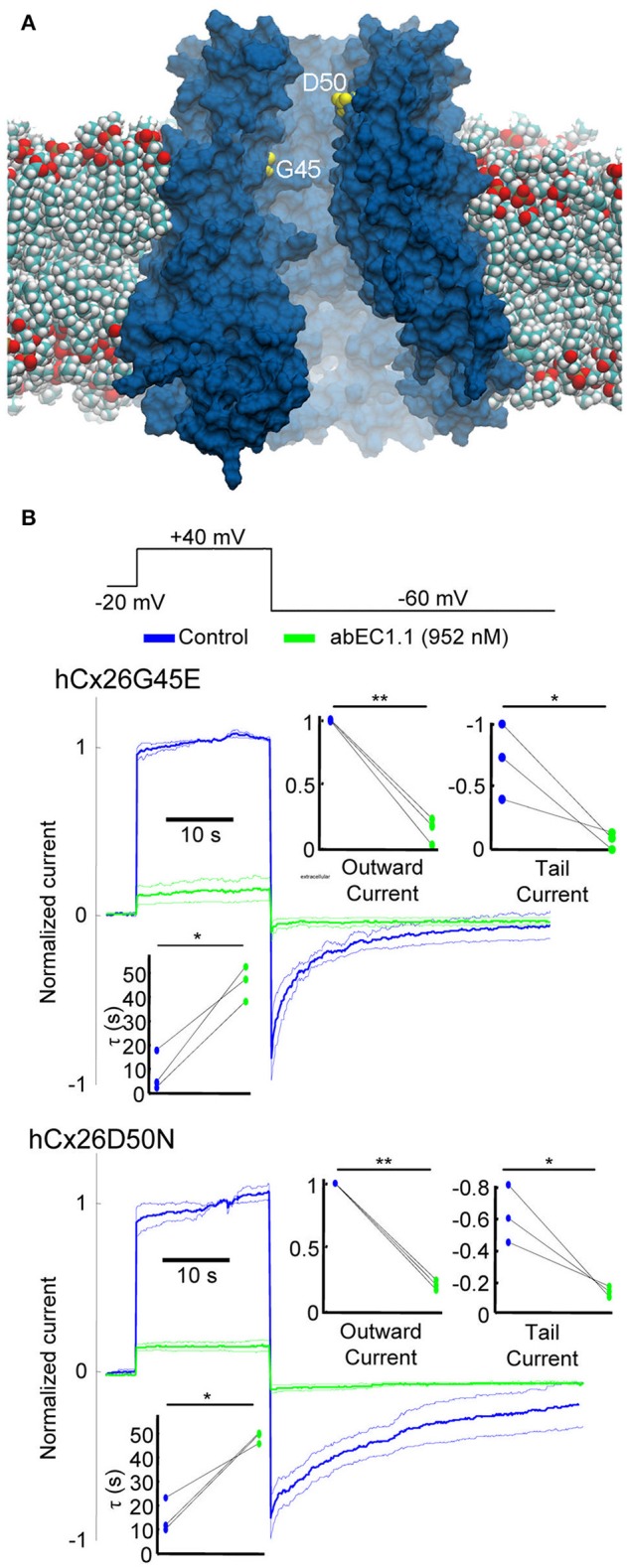
The abEC1.1 antibody reduces the activity of hyperative Cx26 hemichannels in keratinocytes. **(A)** Model of a Cx26 hemichannel embedded in a phospholipid membrane; two of the amino acids implicated in KID/HID syndrome (G45, D50) are shown in yellow. **(B)** Representative whole cell currents elicited by shown voltage commands (black traces) applied to HaCaT cells expressing Cx26 mutants G45E or D50N. Data are mean values (thick traces) ± s.e.m (thin traces) for *n* = 3 cells in each set. Top insets show normalized current immediately after the voltage commands to +40 mV (outward current) and −60 mV (tail current). Bottom inset shows time constant for a single exponential fit to the tail current decay over time. Asterisks indicate significance level between control condition and antibody application (^*^P < 0.05; ^**^P < 0.01, paired *t*-test).

The D50N mutation of Cx26 causes the most common form of KID/HID, whereas the G45E mutation has been linked to lethal forms of the syndrome (Martin and van Steensel, 2015). We induced transient expression of G45E or D50N mutant hemichannels in HaCaT cells. As expected, most cells displayed visible sign of stress within few hours post transfection. Nonetheless, we succeeded to perform patch clamp experiments on some of them. In 0.2 mM extracellular Ca^2+^, Cx26 G45E hemichannel currents were similar to wild type Cx26 currents, whereas Cx26 D50N exhibited faster tail current kinetics, as previously reported for hemichannels expressed in Xenopous oocytes (Sanchez et al., 2013). Importantly, both G45E and D50N, continued to show inhibition by abEC1.1 (Figure 8).

### Identification of residues that are critical for abEC1.1 binding to Cx26 hemichannels

**Figure 9:**
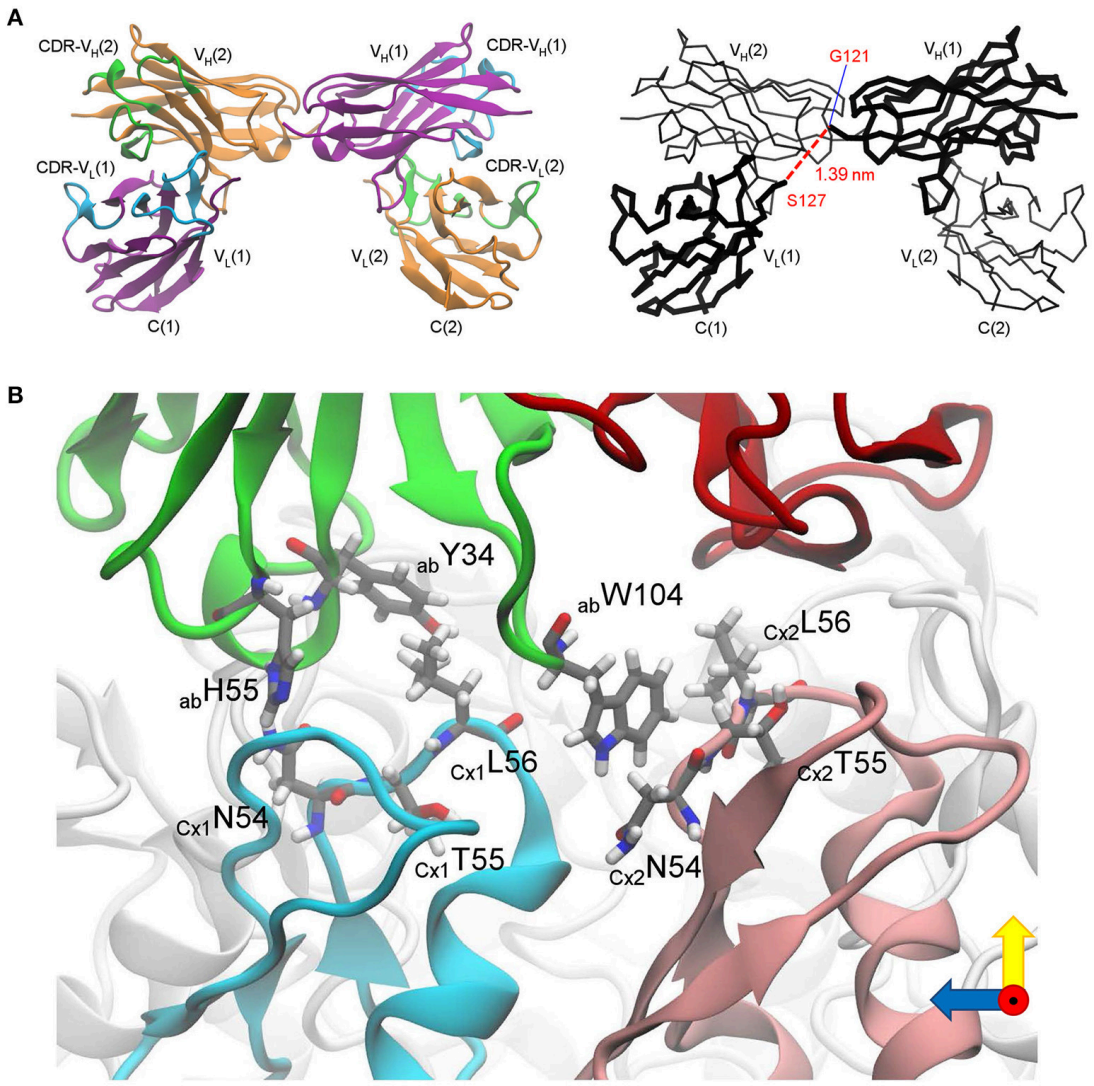
Critical determinants of abEC1.1 antibody binding to Cx26 hemichannels. **(A)** Structure of the scFv domain of abEC1.1, determined by X-ray crystallography. Left: two V_L_-V_H_ pairs are shown in cartoon representation; one polypeptide is drawn in purple with complementarity-determining regions (CDRs) in cyan, the other in orange with CDRs in green. Right: alpha-carbon backbone; chain V_L_(1)-V_H_(1) is drawn in thick lines and V_L_(2)-V_H_(2) in thin lines; the red dashed line between G121 and S127 (1.39 nm) represents 5 of the 7 amino acids that were not solved between V_L_(1) and V_H_(1) (the corresponding line between V_L_(2) and V_*H*_(2) is not shown). **(B)** Residues critical for binding singled out *in silico*: green, antibody (ab) heavy chain (V_H_); red, antibody light chain (V_L_); two protomers of the Cx26 hemichannel (CxA, CxB), which interact with V_H_, are colored in cyan and pink, while the other protomers are colored in light gray; amino acids mentioned in the main text (Y34, H55, W104 for the antibody; N54, T55, L56 for the two connexins) are shown in licorice representation.

Considering abEC1.1 a promising candidate, we solved the crystal structures of its scFv domain (Figure 9A; the statistics of data collection and structure refinement are summarized in Table 1). Five of the seven amino acids that link V_H_ to V_L_ were not solved, however the structure is compatible with that of a diabody (Holliger et al., 1993; Perisic et al., 1994). Therefore, abEC1.1 is expected to assemble as diabody-Fc, i.e a dimer of two scFv-Fc polypetides where V_H_ and V_L_ from one scFv, pair with the complementary domains of a second scFv (Wu et al., 2001).

**Table 1:** Statistics of data collection and scFv structure refinement.

**DIFFRACTION DATA**	
Wavelength (Å)	1.000
Space group	P42_1_2
**CELL PARAMETERS**	
a, b, c (Å)	119.49, 119.49. 94.11
Resolution (Å)	50.00-2.65 (2.70–2.65)
Redundancy	6.6 (4.0)
Completeness (%)	96.1 (92.4)
Average I/σ < I >	10.9 (1.9)
R_merge_ (%)	10.1 (36.7)
**REFINEMENT**	
R_work_/R_free_	0.242/0.267
rmsd bond length (Å)	0.007
rmsd bond angle (Å)	1.137
Average B (Å^2^)	41.46
**RAMACHANDRAN PLOT (%)**	
Favored	98.9
Allowed	1.1
Disallowed	0.0

To gain insight into the interaction of the antibody with its target, we combined the crystal structure of the scFv domain (Figure 9A) with an atomistic model of the Cx26 hemichannel (Zonta et al., 2012). In agreement with the results of Figure 2A, we simulated the docking two scFv diabodies to the extracellular domain of the hemichannel and used molecular dynamics to thermalize the interacting systems. In this computational model, three CDR residues of the abEC1.1 V_H_ (Y34 in CDR1, H55 in CDR2, and W104 in CDR3) interacted with N56, T57, and L58 residues of Cx26 at the apex of the EC1 loop (Figure 9B and Supplementary Video 3).

**Figure 10:**
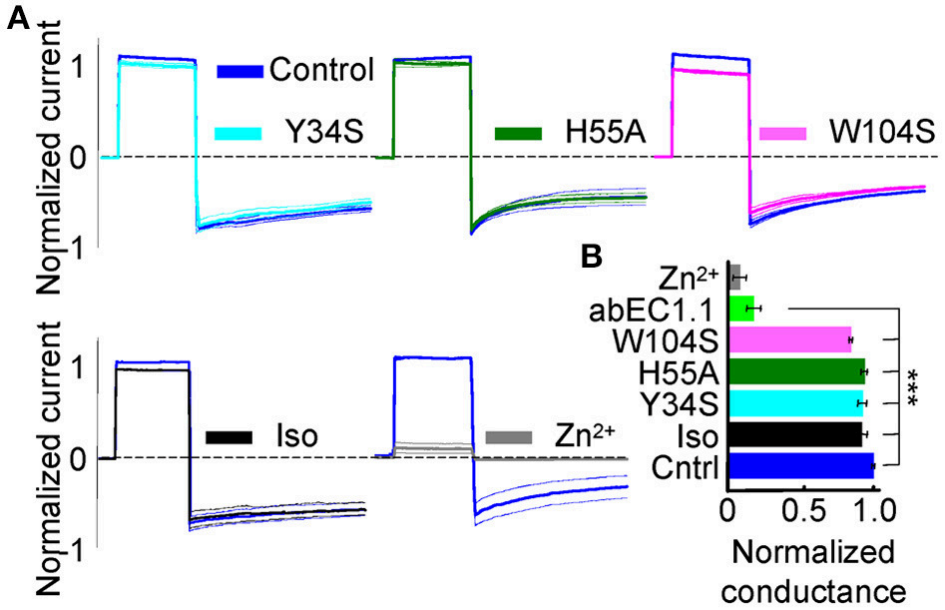
Effect of wild type abEC1.1, its mutants Y34S, H55A, W104S, and an isotype control antibody (Iso) on Cx26 hemichannel currents in HeLa DH transfectants. **(A)** Shown are mean values (thick traces) ± s.e.m. (thin traces) for *n* = 3 cells in each data set. All antibodies were applied by pressure at 952 nM concentration from a glass micropipette; Zn^2+^ was delivered through the perfusion at 0.1 mM concentration. **(B)** Membrane conductance (mean ± s.e.m.) from data in **(A)**, normalized to pre-antibody application levels, was tested using the voltage protocol of Figure 2. Asterisks indicate significance level (^***^P < 0.001, ANOVA) relative to abEC1.1 effect on Cx26 (from Figure 2). Note that antibody residues mentioned in this article do not follow the Kabat numbering scheme.

**Figure 11:**
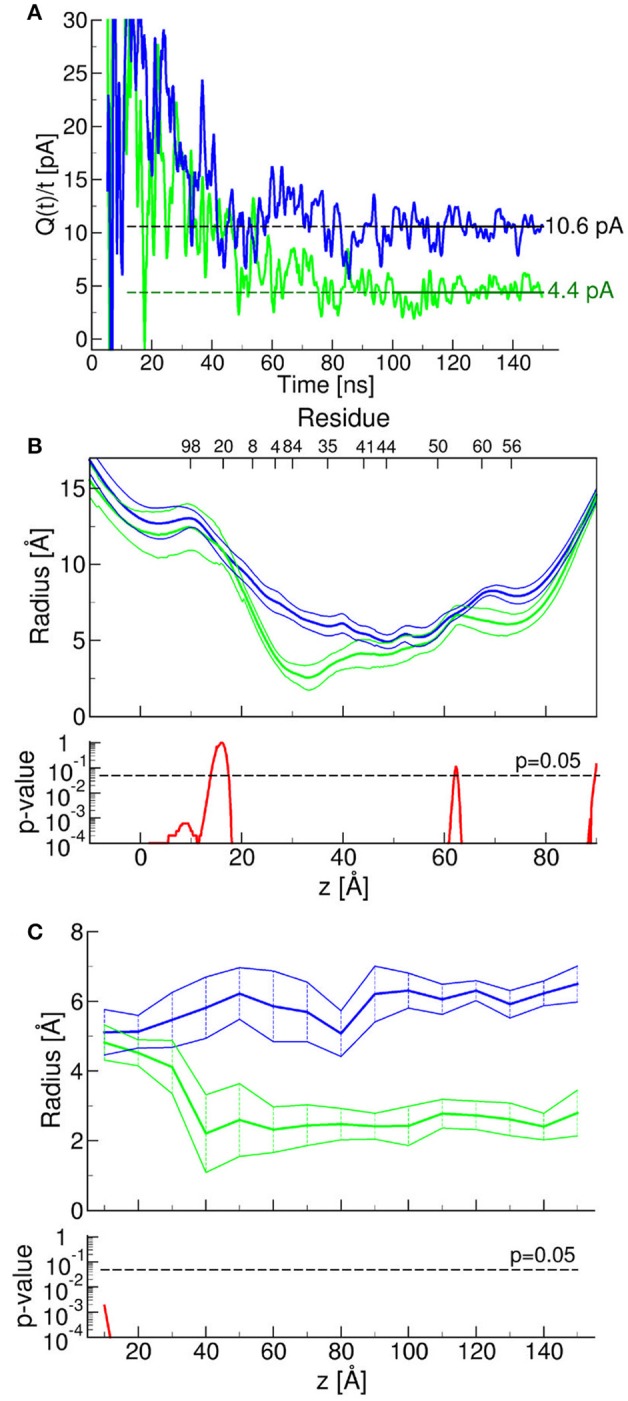
Molecular dynamics simulation of antibody effects on Cx26 hemichannels. Results obtained in the presence or absence of two bound antibodies are represented by green and blue traces, respectively. **(A)** Simulated electrophysiology experiments; for these simulations we applied an external constant electric field *E*_z_ = 0.0022 V/nm, which corresponds to a membrane potential of *V* = *E*_z_*L*_z_ = 40 mV, where *L*_z_ represent the size of the simulation box in the z (i.e., the field) direction (Gumbart et al., 2012). The two traces represent the time course of a function the limit of which, for *t* approaching infinity, is the unitary hemichannel current. The limits, estimated by computing the average of function Q(*t*)/*t* over the last 50 ns of the corresponding molecular dynamic simulation, when the function fluctuated around a steady plateau level (shown in the graph as horizontal lines) were 10.6 ± 1.1 pA (in the absence of antibodies) and 4.4 ± 1.0 pA (in the presence of the antibodies) (*P* < 10^−4^). To reduce the large statistical errors due to the limited time windows, we computed the current with and without antibodies as the averages over the last 50 ns of the functions *Q*(*t*)*/t*, that is after it reaches a plateau. In order reduce the artifacts due to the finite size of the system (Gumbart et al., 2012) we imposed the same box size in both simulations. Next, we computed the ratio between these two averages whereas the error was estimated from the propagation of the standard errors. **(B,C)** Radius of the Cx26 hemichannel pore vs. axial pore coordinate **(B)** or time at *z* = −30 Å **(C)**, where the change imparted by the presence of the bound antibodies is maximal; shown are mean (thick traces) ± s.e.m. (thin traces) with the corresponding *p*-values estimated from the last 10 ns of molecular dynamics simulation. *P*-values were computed with one-tail *t*-tests.

To test this prediction, we mutated one by one the three putatively critical amino acids of abEC1.1. Patch clamp assays confirmed a significantly reduced blocking power of abEC1.1 mutants compared with wild type abEC1.1 (Figure 10). We performed also computer-simulated electrophysiological experiments. To speed up these time-consuming simulations, we removed the V_H_ and V_L_ domains of each bound scFv diabody that did not interact with the hemichannel (Supplementary Video 4). The starting configuration was stable within the simulated time window (150 ns) also in the presence of an external electrical potential. Small monatomic ions escaped through narrow holes at the interface between connexins and bound antibodies, as the latter did not create a perfect seal at the extracellular mouth of the hemichannel. Nevertheless, the bound antibodies reduced the simulated ionic current passing through the pore to 42 ± 14% of its control value (Figure 11A), in qualitative agreement with the experimental results (Figures 2A,B). In this model, the inhibitory effect of the antibodies is associated with a reduction of the hemichannel pore diameter following antibody binding to the EC1 epitope described above (Figures 11B,C). In agreement with these experimental results, molecular dynamics simulations showed that ATP escapes from the extracellular pore vestibule of the free hemichannel in few nanoseconds, whereas bound antibodies impede ATP release to the extracellular milieu (Supplementary Video 5).

Altogether, these results indicate that abEC1.1 inhibits connexin hemichannels by binding an epitope at the apex of the EC1 loop.

## Discussion

By screening a phage library, we successfully isolated a human scFv that binds a peptide derived from the EC1 loop of Cx26, and converted it into the scFv-Fc format (Bujak et al., 2014). The latter offers several advantages over the phage display-derived scFv, including bivalent binding and longer half-life, and may be used in the clinic for therapeutic application (Beck and Reichert, 2011). However, antibodies selected on the basis of binding to a recombinant membrane protein or one of its domains may not bind the same protein when it is in its native context (Lipes et al., 2008). We provide compelling evidence that connexin hemichannels are the identified target of abEC1.1, as opposed to some off-target interaction.

First of all, abEC1.1 inhibited ionic currents through Cx26 hemichannels with IC_50_ of ~40 nM. Furthermore, variants of abEC1.1 carrying single amino acid substitutions in critical V_H_ CDR positions, predicted to mediate the interaction with the Asparagine-Threonine-Leucine (N-T-L) amino acid motif at the apex of the EC1 loop, reduced significantly the ability of abEC1.1 to inhibit Cx26 hemichannel currents in HeLa DH transfectants.

abEC1.1 interfered not only with ionic conductance but also with the release of ATP, as demonstrated by our experiments with Cx26-expressing human keratinocyte-derived cells.

In addition, the antibody inhibited ATP-dependent spontaneous Ca^2+^ signaling activity (Tritsch et al., 2007; Schutz et al., 2010; Rodriguez et al., 2012; Wang and Bergles, 2015; Johnson et al., 2017) in the greater epithelial ridge of cochlear organotypic cultures obtained from P5 mice.

Of note, the results of dye transfer assays in cochlear tissue suggest that the fence function exerted by tight junctions (Rodriguez-Boulan and Nelson, 1989), which provide a boundary between the apical and basolateral plasma membrane domains to maintain cell polarity (Wilcox et al., 2001), was preserved in the superficial layer of non-sensory cells in these organotypic cultures.

Our atomistic model suggests that the inhibitory action of abEC1.1 is mediated by partial occlusion of the extracellular pore mouth and reduction of the hemichannel diameter. The difference between the residual current determined experimentally (18%) and that predicted by the model (42%) may be due the fact that large permeating molecules, not included in our ionic current simulations (e.g., ATP), remain stuck in the antibody-capped hemichannel, further reducing the residual ionic conductance. Despite these shortcomings, the model allowed us to identify critical residues in the binding interface. Thus, we are confident that the *in silico* analysis we performed here can aid antibody refinement by intelligent CDR editing, aimed at improving selectivity and performance, as well as at identifying new antibodies against different connexins.

Last, but not least, we show that abEC1.1, at sub-micromolar concentrations, inhibits Cx26 mutant hemichannels (G45E, D50N) expressed in a human keratinocyte cell line. Thus, these results are remarkable and promising, in a therapeutic perspective, as: (i) is abEC1.1 is a human monoclonal antibody, (ii) the D50N mutation causes the most common form of KID/HID syndrome, whereas (iii) the G45E mutation has been linked to lethal forms of the syndrome (Martin and van Steensel, 2015).

Recently, mefluoquine and Zn^2+^ have been reported to inhibit these and other mutants of Cx26 with IC_50_ of 16 and 3 μM, respectively, but both required concentrations of the order of 100 μM to eliminate >70% of hemichannel currents (Sanchez et al., 2014; Levit et al., 2015). Mefluoquine is known to block voltage-gated L-type calcium channels, Kir6.2 and KvLQT1 potassium channels and pannexin channels, whereas Zn^2+^, as most divalent cations, is likely to promote numerous off-target interactions (Levit et al., 2015). Furthermore, we showed here that mefluoquine, unlike abEC1.1, is toxic to cultured human keratinocytes as well as mouse cochlear tissue.

*Ex vivo* gene therapy (Mavilio et al., 2006; De Luca et al., 2009), stem cell transplantation (Tolar et al., 2009; Tamai et al., 2011), and administration of allogeneic fibroblasts (Wong et al., 2008) or recombinant protein (Remington et al., 2009) have been explored as potential therapeutic avenues for patients with inherited skin disorders. However, these approaches meet both technical and safety problems (Uitto et al., 2012).

Intradermal injection of monoclonal neutralizing antibodies (FnAbs) against actin-remodeling protein has been reported to accelerate wound reepithelialization improving the macroscopic appearance of early scars in porcine models of wound healing (Jackson et al., 2012). Likewise, topical application of an antibody cream formulation significantly improved the skin barrier function and reduced the skin fragility (up to 50%) in a murine model of epidermolysis bullosa acquisista (Kopecki et al., 2013). Further studies will clarify whether the antibody we identified can be used to treat connexin-related skin pathologies in animal models.

## Data availability

The crystal structure and the amino acid sequence of the abEC1.1 scFv domain have been deposited in the Protein Data Bank (PDB) under the accession codes 5WYM.

## Code availability

Computer code used to analyze molecular dynamics data is available from the authors.

## Author contributions

FM, GY, and FZ designed the studies; FM, GY, and RAL provided resources to conduct the studies; FM, GY, FZ, ZQ, and AMS supervised the work or junior colleagues; MR and FS were in charge of animal welfare and performed quality controls; LX, AC, PM, SL, DB, FC, GC, VZ, GZ, FB, CN, CP, FlM, YC, SX, XY, JL, XL, WW and SW performed the experiments. DB and FZ performed molecular dynamics simulations. FM wrote the manuscript; FZ and AMS edited the text.

### Conflict of interest statement

RAL is a founder of Zebra Biologics Inc.; GY is a partner of Zebra Biologics Inc. The other authors declare that the research was conducted in the absence of any commercial or financial relationships that could be construed as a potential conflict of interest.
