# Supplementary material for: Design and Characterization of a Human Monoclonal Antibody that Modulates Mutant Connexin 26 Hemichannels Implicated in Deafness and Skin Disorders
Source: Front Mol Neurosci. 2017 Sep 22;10:298. doi: 10.3389/fnmol.2017.00298 (PMC5615210; doi:10.3389/fnmol.2017.00298)
Supplement: Supplementary file 6 [file Presentation1.PDF]

VIDEO DESCRIPTIONS

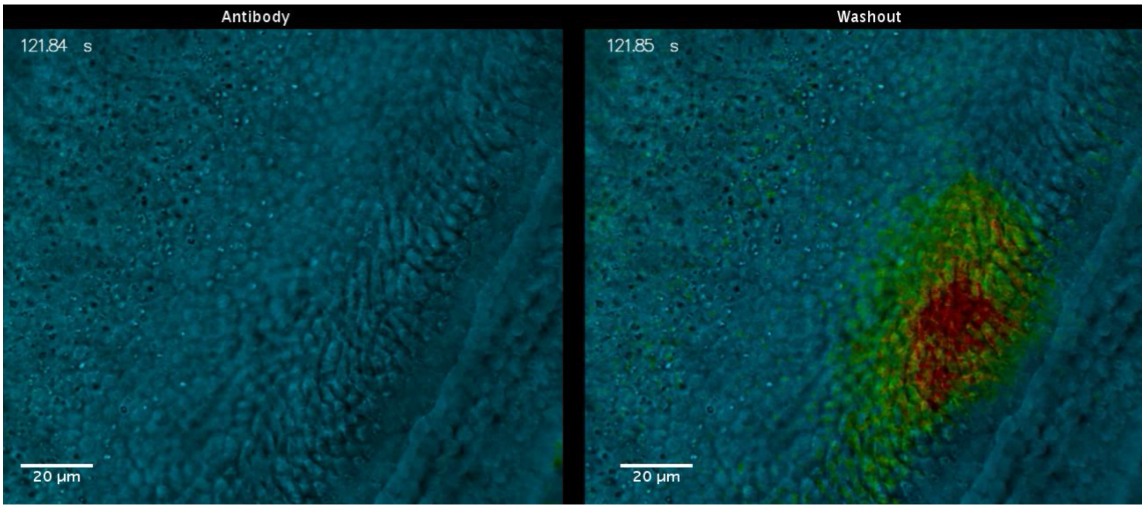

Video 1. Fluo-4 fluorescence change ($\Delta F/F_0$) recorded in an apical turn culture from the GER of a P5 mouse imaged at 1 frame per second in the presence of the abEC1.1 antibody (left) and after antibody washout with normal extracellular solution (right); pseudo-color scale as in Figure 5a.

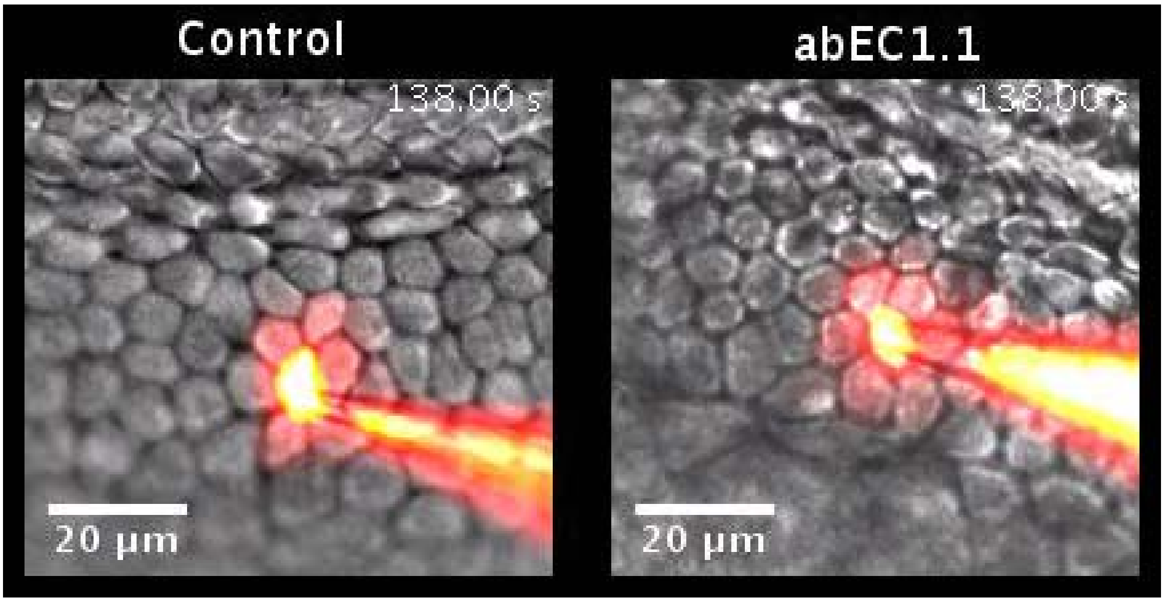

Video 2. Lucifer yellow transfer assay, performed in the absence of antibody (left) and after 4 hour incubation with abEC1.1 (952 nM, right).

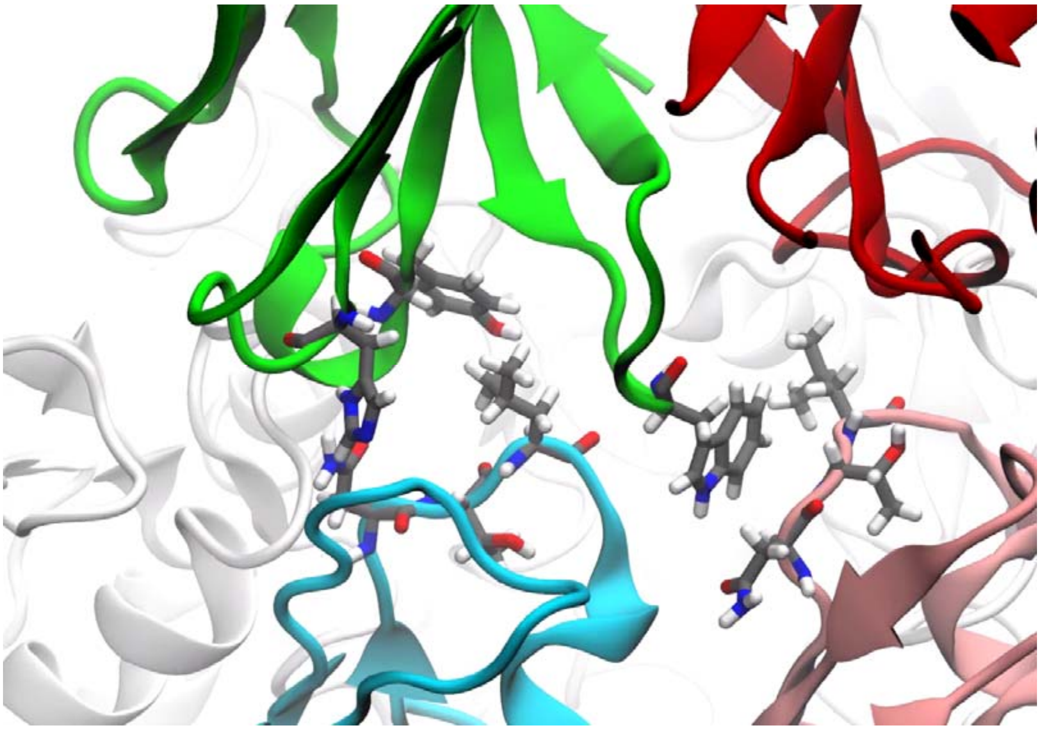

Video 3. Critical residues implicated in abEC1.1 antibody binding to the hCx26 hemichannel.

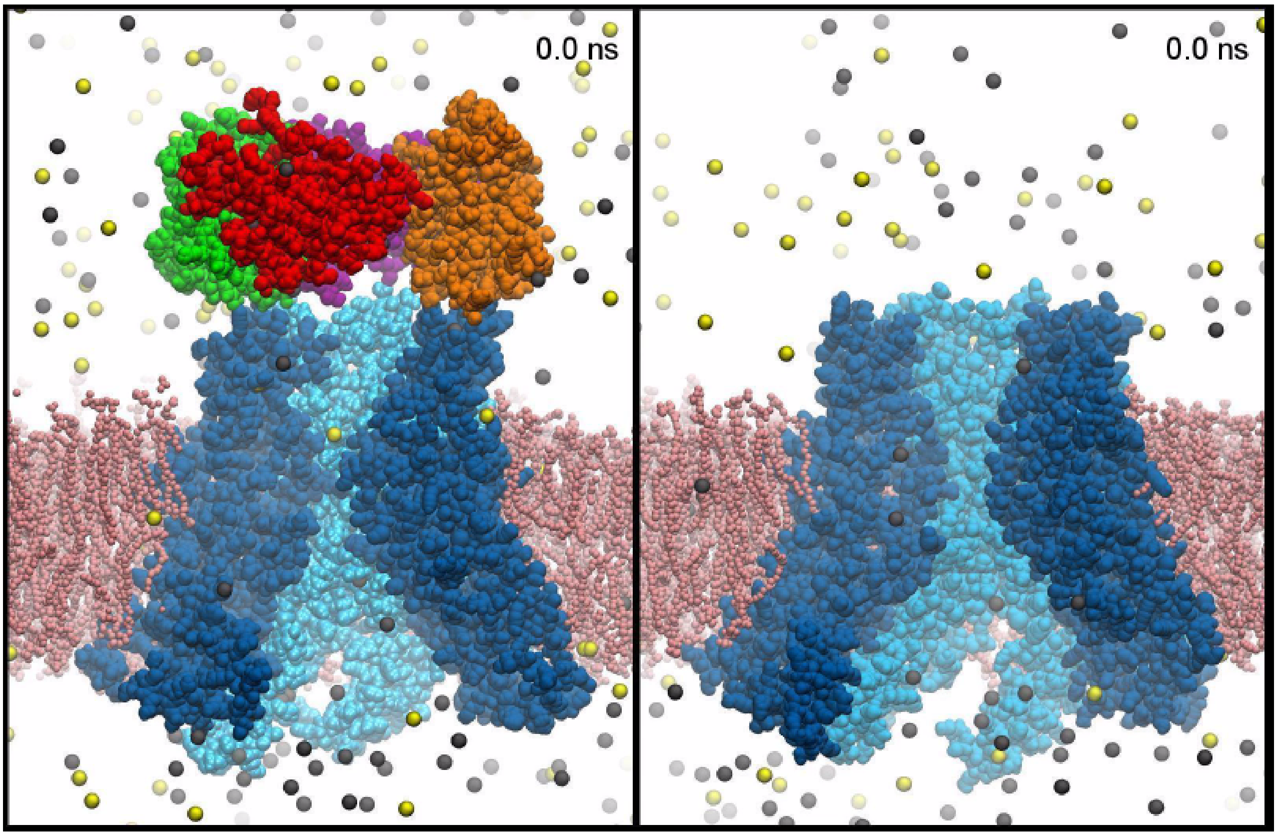

Video 4. Molecular dynamics simulations showing the effect of the abEC1.1 antibody on ion transit through a hCx26 hemichannel.

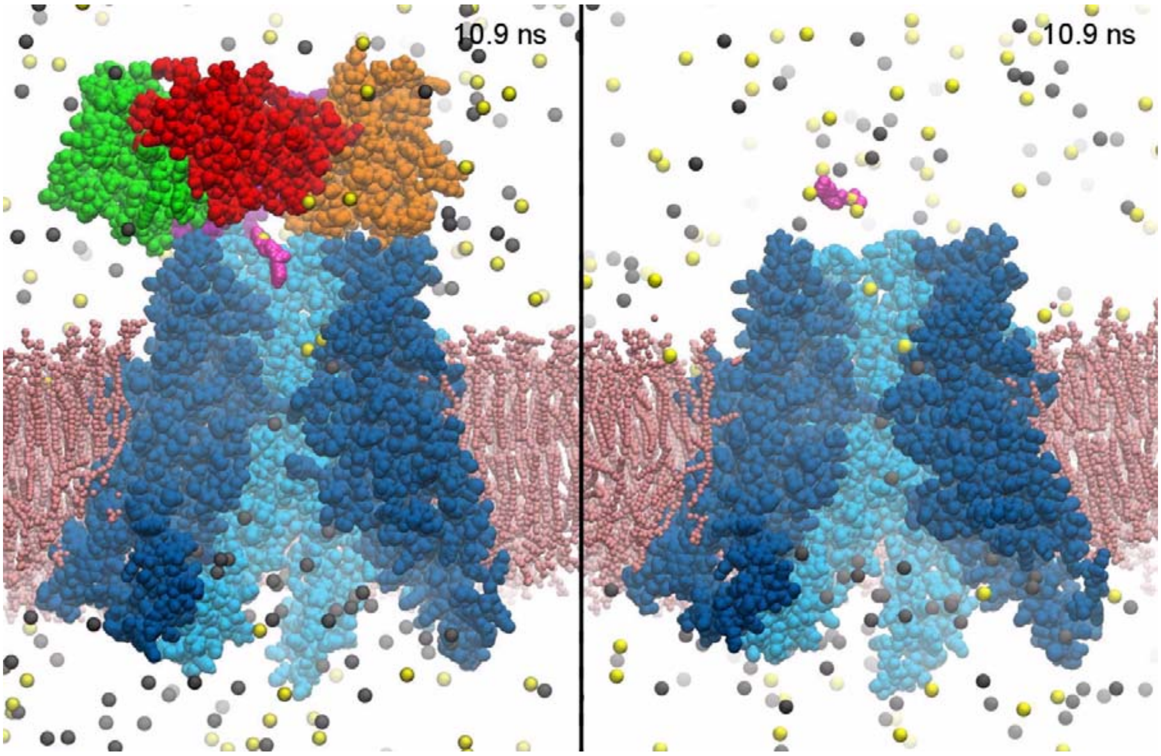

Video 5. Molecular dynamics simulations showing that, in the presence of two bound scFvs of the abEC1.1 antibody, ATP release from a hCx26 hemichannel is impeded.

Methods for Video 5: To simulate the effect of abEC1.1 on the permeation of ATP, we introduced a single ATP molecule in the extracellular vestibule in the configuration with two docked antibodies and simulated its dynamics in the presence of an external field and NTV ensemble. Potentials as large as 120 mV were unable to force the passage of the ATP molecule through the reduced residual opening between docked antibodies and connexins in the simulated time frames (around 100 ns).
